# Textured Asymmetric Membrane Electrode Assemblies of Piezoelectric Phosphorene and Ti_3_C_2_T_x_ MXene Heterostructures for Enhanced Electrochemical Stability and Kinetics in LIBs

**DOI:** 10.1007/s40820-023-01265-5

**Published:** 2024-01-08

**Authors:** Yihui Li, Juan Xie, Ruofei Wang, Shugang Min, Zewen Xu, Yangjian Ding, Pengcheng Su, Xingmin Zhang, Liyu Wei, Jing-Feng Li, Zhaoqiang Chu, Jingyu Sun, Cheng Huang

**Affiliations:** 1 Volta and DiPole Materials Labs, College of Energy, Soochow Institute for Energy and Materials InnovationS (SIEMIS), Key Laboratory of Advanced Carbon Materials and Wearable Energy Technologies of Jiangsu Province, Key Laboratory of Core Technology of High Specific Energy Battery and Key Materials for Petroleum and Chemical Industry, Soochow University, Suzhou, 215006 People’s Republic of China; 2 School of Materials Science and Engineering, Jiangsu University, Zhenjiang, 212013 People’s Republic of China; 3 College of Underwater Acoustic Engineering, Harbin Engineering University, Harbin, 150001 People’s Republic of China; 4 Shanghai Synchrotron Radiation Facility, Shanghai Advanced Research Institute, Shanghai Institute of Applied Physics, Chinese Academy of Sciences, Shanghai, 201204 People’s Republic of China; 5 State Key Laboratory of New Ceramics and Fine Processing, School of Materials Science and Engineering, Tsinghua University, Beijing, 100084 People’s Republic of China; 6 High Density Materials Technology Center for Flexible Hybrid Electronics, Suzhou Institute of Electronic Functional Materials Technology, Suzhou Industrial Technology Research Institute, Suzhou, 215151 People’s Republic of China; 7 Institute of Advanced Materials and Institute of Membrane Science and Technology, Jiangsu National Synergistic Innovation Center for Advanced Materials, Suzhou Laboratory and Nanjing Tech University, Nanjing, 211816 People’s Republic of China

**Keywords:** Phosphorene, Nanopiezocomposite, Piezo-electrochemical coupling, Membrane electrode assembly, Lithium-ion storage

## Abstract

Black phosphorus with a superior theoretical capacity (2596 mAh g^−1^) and high conductivity is regarded as one of the powerful candidates for lithium-ion battery (LIB) anode materials, whereas the severe volume expansion and sluggish kinetics still impede its applications in LIBs. By contrast, the exfoliated two-dimensional phosphorene owns negligible volume variation, and its intrinsic piezoelectricity is considered to be beneficial to the Li-ion transfer kinetics, while its positive influence has not been discussed yet. Herein, a phosphorene/MXene heterostructure-textured nanopiezocomposite is proposed with even phosphorene distribution and enhanced piezo-electrochemical coupling as an applicable free-standing asymmetric membrane electrode beyond the skin effect for enhanced Li-ion storage. The experimental and simulation analysis reveals that the embedded phosphorene nanosheets not only provide abundant active sites for Li-ions, but also endow the nanocomposite with favorable piezoelectricity, thus promoting the Li-ion transfer kinetics by generating the piezoelectric field serving as an extra accelerator. By waltzing with the MXene framework, the optimized electrode exhibits enhanced kinetics and stability, achieving stable cycling performances for 1,000 cycles at 2 A g^−1^, and delivering a high reversible capacity of 524 mAh g^−1^ at − 20 ℃, indicating the positive influence of the structural merits of self-assembled nanopiezocomposites on promoting stability and kinetics.

**Supplementary Information:**

The online version contains supplementary material available at 10.1007/s40820-023-01265-5.

## Highlights

- An asymmetric membrane electrode based on phosphorene/MXene heterostructure-textured nanopiezocomposite was fabricated via a polar urea-assisted self-assembly strategy and additive manufacturing of the heterostructure beyond the skin effect.
- The merits of this novel asymmetric heterostructure-textured electrode and its intrinsic piezoelectricity were detailedly discussed.
- The stepwise lithiation process of phosphorene was revealed, and the enhanced electrochemical properties of this phosphorene-based nanopiezocomposite textured electrode were verified by the improved cycling stability and kinetics.

**Supplementary Information:**

The online version contains supplementary material available at 10.1007/s40820-023-01265-5.

## Graphical abstract

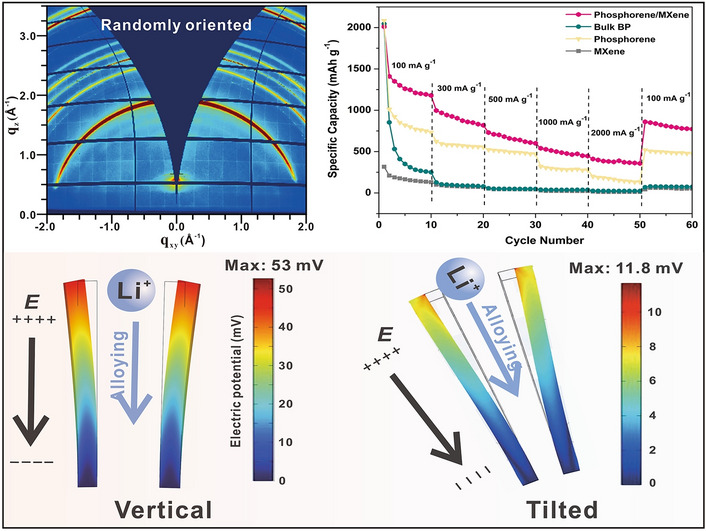

## Introduction

The depletion and severe pollution of fossil fuels quicken the pace of pursuing the clean and renewable energy sources [1]. Nowadays, a series of efficient energy storage systems have been developed, such as alkali-metal-ion batteries [2, 3], dual-ion batteries (DIBs) [4, 5], aqueous zinc-ion batteries (AZIBs) [6], redox electrolytes-assisting AZIBs [7], aqueous supercapacitors (SCs) [8], Li/Na-ion capacitors (L/NICs) [9, 10], supercabatteries (SCBs) [11–13] and dual-ion supercabatteries (DICBs) [14]. Among those promising energy storage devices, lithium-ion batteries (LIBs) are regarded as one of the most competitive candidates by virtue of its high energy density and comparatively low cost. However, the typical graphite anode only delivers a theoretical capacity of 372 mAh g^−1^, making it hard to fulfill the growing demands of electronic devices [15], and furthermore its poor rate capability and inferior kinetics might restrict its applications in severe conditions such as low-temperature and/or fast charging environment [16]. Therefore, developing high-rate and high-capacity anode materials still becomes the main concern of the researchers [17], and a series of potential anode materials with superior theoretical capacities such as Si (3572 mAh g^−1^) [18], P (2596 mAh g^−1^) [19] and Ge (1600 mAh g^−1^) [20], have been explored in the last few years. Among those promising anode materials, phosphorus possesses the highest average charge/discharge potential than that of Si and Ge, which is in favor of avoiding the risk of lithium plating at high current density [21]. In addition, phosphorus tends to form covalent bonds with other inorganic materials (such as P–C bond) and maintains good electrical connection during the lithiation process, making it available to collaborate with other conductive substrates to fully realize its potential. Among the three common allotropes of phosphorus, BP exhibits much higher conductivity (~ 10^2^ S cm^−1^) [22] and superior lithium-ion kinetics (hundreds of times theoretically faster than that of other conventional 2D materials such as graphene and MoS_2_) [23], making it possible to be considered as the promising anode material of LIBs. However, black phosphorus (BP) anode suffers severe volume variation (about 300%) during the lithiation/delithiation processes (which is similar to that of Si anode) [24], the structural deformation could lead to the delamination and electrical separation between the active materials and current collector, thereby increasing the resistance and damaging the cycling stabilities. In addition, the expansion of BP powder could also destroy its intrinsic layered structure and trim back the number of exposed active sites, causing the irreversible capacity loss. Though several structural optimization strategies were proposed to address these drawbacks [25, 26], the swelling of BP-based electrodes could not be settled thoroughly but bated, as long as it remains bulk structure.

Inspired by the exploration and utilization of 2D graphene which is derived from layered graphite, the unique properties of single-layer or few-layer BP (phosphorene) such as the tunable bandgap grab the attention of researchers [27–29]. Moreover, it is suggested that the volume variation could be effectively eliminated and more Li-ion channels could be dug when the bulk BP material is exfoliated to phosphorene or black phosphorus quantum dots (BPQDs) via liquid phase exfoliation (LPE) method, thus greatly improving its electrochemical properties [30]. By leveraging this efficient method, researchers can fabricate high-quality phosphorene and further squeeze the values of those phosphorene-based anodes in alkali-ion batteries [31, 32]. In addition, the exfoliated phosphorene possesses desirable piezoelectricity [33], which is expected to enhance the kinetics of alkali-ions by the piezo-electrochemical coupling effect and the generated self-built-in piezoelectric field [34–36]. However, the restacking and agglomeration of those exfoliated phosphorene are still unavoidable during the slurry-coating procedures of the universal electrode fabrication route, resulting in the inferior piezoelectricity and conspicuous capacity loss.

Compared to the conventional slurry-coating electrode, the self-assembled free-standing membrane electrode possesses the better controllability of the orientation of 2D active material and electrode structure. To avoid the uneven distribution and agglomeration of phosphorene, several phosphorene-based membrane electrode assemblies (MEA) were fabricated by researchers. For instance, Chen et al. [37] and Liu et al. [38] prepared free-standing phosphorene/graphene MEA and sandwiched phosphorus/graphene MEA, respectively, and those MEA exhibited favorable electrochemical properties. In general, in consideration of the electrochemical kinetics and structural stability of MEA, neither the 0° polarization orientation (flat) nor the 90° polarization orientation (vertical) possesses the optimal properties, and the tailored 0^°^–90° polarization modulation may exhibit the optimal electrochemical and electromechanical properties [39]. Similarly, piezoelectric materials with tailored 0^°^–90° polarization orientation modulation may possess the much better piezoelectric and electromechanical performances [40, 41]. Therefore, rationally tailoring the anisotropic polarization of phosphorene with a tilted facet is critical to retain its piezoelectricity and enhance the electrochemical kinetics and stability, whereas it still remains a challenge. Furthermore, though BP possesses the higher lithiation potential than graphite, the risk of lithium plating still exists under fast-charging condition, hence the structure regulation of phosphorene/MXene MEA also contributes to the inhibition of lithium plating and dead lithium under fast-charging conditions [42].

Herein, a heterostructure-textured asymmetric MEA of piezoelectric phosphorene and MXene nanoarchitecture was developed through the multifunctional urea-assisted orientation and cross-linking strategy beyond the skin effect, and the proposed phosphorene/MXene nanopiezocomposite could be directly used as the LIB anode. The advantages of the proposed phosphorene-based MEA are fourfold: (1) the addition of polar urea molecules could promote the uniform distribution of those exfoliated phosphorene and prevent them from aggregation during the self-assembly process, thus effectively suppressing the volume variation and improving cycling stabilities. (2) This nanopiezocomposite favorably inherits the intrinsic piezoelectricity of phosphorene and the self-built-in piezoelectric field could be generated during the lithiation process, thus greatly enhancing the Li-ion kinetics by serving as the extra accelerator. (3) As the majority of the MEA, the highly conductive Ti_3_C_2_T_x_ framework endows the MEA with favorable overall conductivity, resulting in the low transfer resistances. In addition, those few-layered Ti_3_C_2_T_x_ nanosheets can provide numerous channels for Li-ion to transfer and shorten the migration routes of electrons and Li-ions. (4) This free-standing MEA could be fabricated through the facile filtration and freeze-drying procedures without the addition of binder or current collector, thus avoiding the possible aggregation of phosphorene during the conventional slurry fabrication process and further improving the overall energy density. By virtue of the merits mentioned above, the optimal phosphorene/Ti_3_C_2_T_x_ MEA exhibited the enhanced electrochemical properties with highly reversible capacity (1463 mAh g^−1^ at 100 mA g^−1^) and cycling stability (420 mAh g^−1^ for 1,000 cycles at 500 mA g^−1^). Moreover, a high reversible capacity of 524 mAh g^−1^ could be reached at − 20 ℃, indicating its great potential in low-temperature environment.

## Experimental and Calculation

### Materials

The bulk BP powders and *N*-methyl-2-pyrrolidone (NMP) solvent were purchased from Sigma-Aldrich, the MXene precursor Ti_3_AlC_2_ was obtained from Alfa Aesar, and the lithium fluoride (LiF), urea and hydrochloric acid (HCl, 12 M) were acquired from Sinopharm Chemical Reagent Company. The commercial LiFePO_4_ (LFP) powder was bought from Hefei Kejing Materials Technology Company. All the reagents are analytical grade and adopted without any further treatments.

### Fabrication of Phosphorene Nanosheets

Typically, 0.5 g bulk BP powder was dispersed into the 100 mL NMP solvent via ultrasonication treatment for 6 h. The overall exfoliation processes were conducted under the protection of an ice bath and argon atmosphere to prevent the oxidation of BP. The obtained solution was then centrifugated at 1500 rpm for 30 min to remove the unexfoliated BP. And the remained solution was again centrifugated at 14000 rpm for 30 min to collect the as-prepared phosphorene. The final phosphorene sample was washed with ethanol and deionized (DI) water for several times (centrifugated at 14000 rpm for 30 min), and redispersed within 50 mL DI water for further use. The specific concentration of the phosphorene solution was ~ 1.5 mg mL^−1^, which could be roughly evaluated by subtracting the dried weight of the removed unexfoliated BP in the first centrifugation from the total mass.

### Fabrication of Few-layered Ti_3_C_2_T_x_ MXene

The conventional mild method was adopted to prepare few-layered MXene [43]. Typically, 2 g LiF was put into 40 mL HCl (9 M) and stirred for 30 min, and then, 2 g Ti_3_AlC_2_ powder was added slowly. After etching for 24 h at 35 ℃, the suspension was filtered and the residue was then washed with DI water for several times through the centrifugation at 3500 rpm until its pH was around 6, and thus, the multilayered MXene was obtained. Subsequently, the ultrasonication treatment was applied for 2 h under the protection of an ice bath and argon atmosphere to prevent the oxidation. The few-layered MXene solution was finally obtained by centrifugation of the solution at 3500 rpm for 30 min. The specific concentration of this solution could be calculated by weighting the mass of dried MXene film which was made of a known volume of as-prepared MXene solution.

### Fabrication of Phosphorene/MXene MEA

Typically, the urea was added into the MXene solution with magnetic stirring for 1 h, and the mass ratio of urea/MXene was 1:15–20. After the dispersion of urea, the phosphorene solution was added dropwise into the above dispersion accompanied by magnetic stirring for 4 h and ultrasonic treatment for 4 h. The argon atmosphere and ice bath were applied for the entire self-assembly processes to prevent oxidation. Then, a vacuum filtration was performed and the Celgard 3501 membrane was chosen as the filter. After a 24-h freeze–drying process, the free-standing phosphorene/MXene nanocomposite MEA was obtained by removing the Celgard 3501 membrane. The nanocomposite MEA with different ratios of phosphorene/MXene was fabricated by adjusting the appropriate volume of phosphorene solution.

### Characterization

The X-ray diffraction (XRD) and in situ XRD patterns of the samples were performed by a Bruker D8 Advance X-ray diffractometer using Cu Kα radiation (*λ* = 1.5418 Å). Raman tests were conducted by a Horiba HR Evolution laser confocal Raman spectrometer (*λ* = 633 nm). The X-ray photoelectron spectroscopy (XPS) spectra were obtained from a Thermo Scientific ESCALAB250 X-ray photoelectron spectrometer to determine the surface chemical bonding states. Bruker dimension icon atomic force microscope (AFM) was hired to evaluate the surface topography and the sample thickness. The scanning electron microscopy (SEM) images and the corresponding energy-dispersive X-ray spectroscopy (EDS) spectra of the films were obtained by a scanning electron microscope (Hitachi SU8010) with an accelerating voltage of 10 kV. The high-resolution transmission electron microscopy (HRTEM) morphology and element mapping of the samples as well as high-angle annular dark-field (HAADF) imaging were collected by a high-resolution filed-emission scanning transmission electron microscope (HRSTEM, JEOL JEM-2100F) with an accelerating voltage of 80 kV. The PL spectrum of as-prepared phosphorene was measured by the Hitachi F-7000 spectrophotometer with an excitation wavelength of 330 nm. The quasistatic piezoelectric coefficients *d*_33_ of the nanocomposite films were collected from PolyK Quasi-Static Piezoelectric Constant *d*_33_ Meter under the dynamic force of 0.25 N. The load–displacement curves and corresponding mechanical modulus and hardness analysis of the film samples were carried out on an instrumented nano-indenter (Agilent, Nanoindenter G200) with a surface approach distance of about 5000 nm. Piezoresponse force microscopy (PFM) was also utilized to characterize the piezoelectric properties of the phosphorene samples. The piezoelectricity of the phosphorene was carried out by using an Asylum Research MPF-3D AFM with a Cr/Pt coated tip. The phosphorene was attached to the appropriate surface of the ITO/PET substrate in order to avoid sliding and electrical breakdown of the phosphorene during the measurements. The resonance frequency of the cantilever is 75 kHz in air, and the spring constant is 3 N m^−1^ (Budgetsensors probes, Multi75E-G, Innovative Solutions Bulgaria Ltd., Sofia, Bulgaria). Tapping mode was employed for all the topography mappings, while the piezoelectric responses were addressed using SS-PFM. During the switching test, the tip was fixed at a certain position, and a DC voltage with a superposition of triangular wave and sawtooth characteristic was applied, and an AC signal was simultaneously applied to measure the amplitudes. Synchrotron-based grazing-incidence X-ray diffraction (GIXRD) tests were conducted to evaluate the improvement of the orientation and alignment of nanoplatelet rearrangements within the self-assembled nanocomposite films. The grazing-incidence wide angle X-ray scattering (GIWAXS) patterns were measured at the BL02U2 surface diffraction beamline of the Shanghai Synchrotron Radiation Facility. The wavelength of the X-ray was 1.24 Å.

### Cell Assembly and Electrochemical Tests

All the electrochemical evaluations were conducted on the typical coin cells (CR-2025) assembled in an argon-filled glove box. The Celgard-2300 polypropylene film was chosen as the separator in both the half-cell and full-cell systems, and the conventional LIB electrolyte for the cell is comprised of 1 M LiPF_6_ solution dissolved within the mixture of ethylene carbonate (EC)/diethyl carbonate (DEC) (*V*_EC:_*V*_DEC_ = 1:1). The free-standing pure MXene film and phosphorene/MXene nanocomposite film were hired as the electrode directly with the diameter of 12 mm, and the average mass loading ranged from 1.5 to 2.0 mg cm^−2^. The phosphorene nanosheets were collected after a freeze–drying process from its solution and served as the active material for the phosphorene electrode. The bulk BP, phosphorene and LFP electrodes were obtained through the slurry coating procedure, which consisted of the active materials, PVDF binder and Super P at the ratio of 80%:10%:10% and then coated on copper foil and aluminum foil, respectively. After being dried at 120 ℃ for 12 h and the rolling press procedure, those electrodes cut with the diameter of 12 mm were obtained. The average mass loadings of bulk BP and phosphorene anodes were about 2.1 and 1.5 mg cm^−2^, respectively, and the mass loading of LFP cathode was designed to possess the low *N*/*P* ratio of 1.2–1.5 relative to the proposed nanocomposite electrode. For the half-cell system, the lithium metal with a diameter of 10 mm was hired as both counter and reference electrode, whereas the full-cell system was comprised of the appropriate LFP cathode and the phosphorene/MXene anode. The CHI660E electrochemical workstation was exploited to perform the cyclic voltammetry (CV) curves over the potential range of 0.005–3 V versus Li/Li^+^ at various scan rates and the electrochemical impedance spectroscopy (EIS) experiments ranged from 0.1 to 100 kHz. The galvanostatic charge–discharge curves were performed by the LAND CT2001A battery test system between the various voltage window (0.005–3 V for half-cell system and 1–3 V for full-cell system) at the specific current densities at room temperature, and the BTC-405 Low-temperature battery test system was applied to collect those discharge/charge curves in various low-temperature environments.

### Simulation and Computational Method

The COMSOL Multiphysics software was hired to simulate the intrinsic piezoelectric potential. The simplified model of phosphorene nanosheet was set to 25 × 3 × 40 (nm) (length × width × height) with the specific parameters (density: 2.69 g cm^−3^; Young modulus: 94 Gpa; dielectric constant: *ε*_*r*_ = 4.2). The Vienna ab initio Simulation Package was employed for all the theoretical calculations on Li binding and diffusion behaviors in different interfaces, including the bilayer black phosphorene (BL-BP) and BP/Ti_3_C_2_T_2_ (T = F or O) heterostructures [44]. The wave-function was described by the projected augmented wave (PAW) method [45]. The exchange–correlation interaction of electrons was treated by Perdew–Burke–Ernzerhof functional within the framework of generalized gradient approximation (GGA-PBE) [46]. A cutoff energy of 400 eV was adopted for the plane-wave basis set. The Grimme’s dispersion correction (DFT-D2) was included to account for the van der Waals interaction. During geometry optimization, the convergence was achieved when energy and force were less than 10^−5^ eV and 0.02 eV Å^−1^, respectively. To minimize the lattice mismatch between BP and Ti_3_C_2_T_x_, we constructed a 3 × 2 supercell of BP and 2 × 3 supercell of orthorhombic Ti_3_C_2_T_2_ to form the BP/Ti_3_C_2_T_2_ heterostructures by stacking them vertically. The Monkhorst–Pack k-point mesh was 3 × 3 × 1 during the calculations. To identify the minimum energy path and diffusion barrier, the climbing image nudged elastic band (CI-NEB) method was applied [47]. The adsorption energy (*E*_ads_) of Li-ion on different substrates was calculated as follows:

1 $$ E_{{{\text{ads}}}} = E_{{{\text{Li}}*}} - E_{{{\text{Li}}}} - E_{*} $$

where *E*_Li*_, *E*_Li_ and E_*_ denote the total energy of Li-ion on the substrate, the energy of Li atom derived from its bulk phase, and the energy of the substrate, respectively, and the smaller *E*_ads_ value means stronger adsorption of Li-ion on the substrate.

## Results and Discussion

### Structural Characterization

**Fig. 1:**
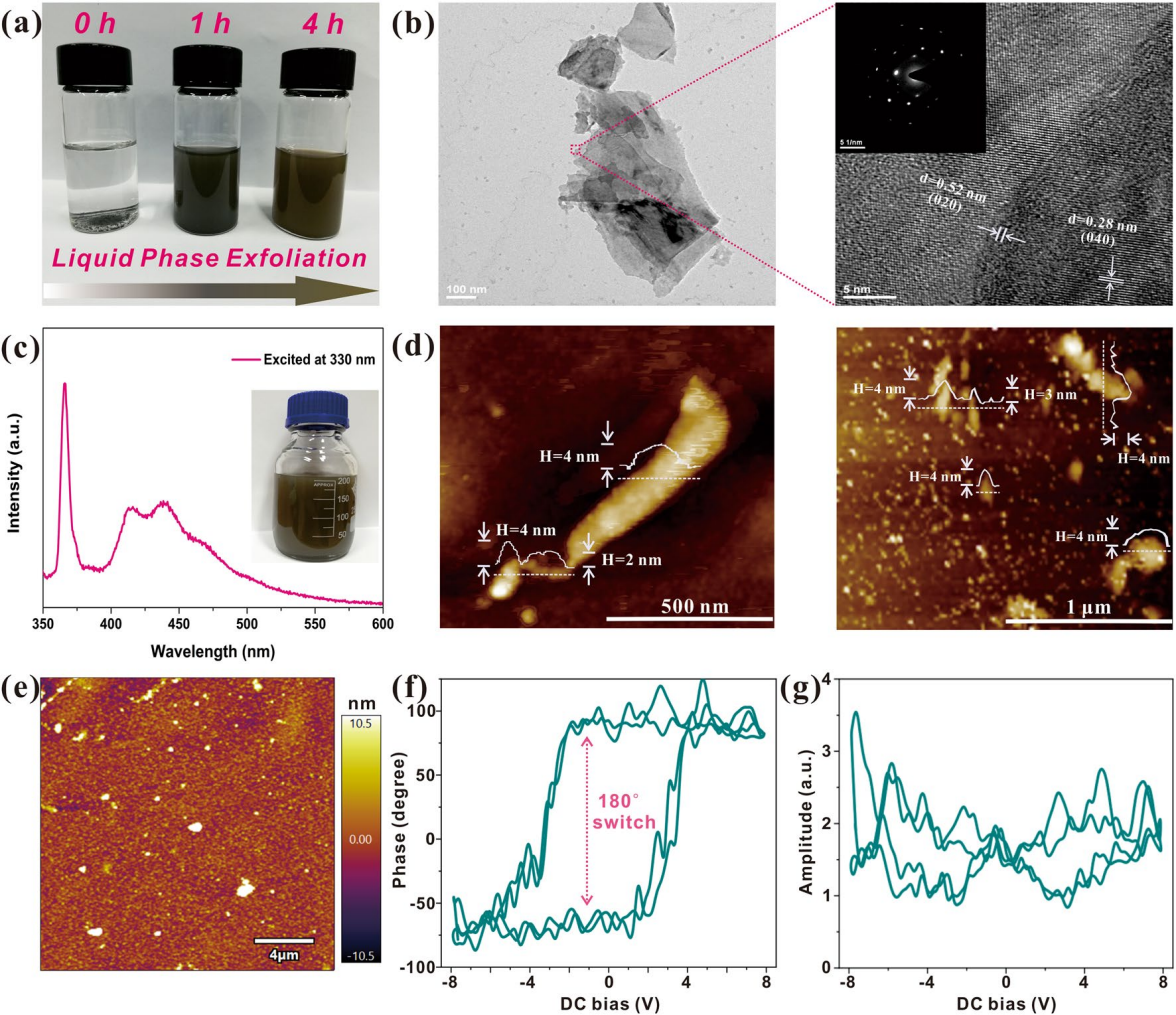
Characterization of proposed phosphorene. **a** Color of phosphorene solution (1 mg mL^−1^ in NMP) with different exfoliation time. **b** TEM and HRTEM images of as-prepared phosphorene (inset shows the electron diffraction pattern). **c** PL spectrum of as-prepared phosphorene and **d** its corresponding AFM images. **e** Test area of PFM. **f** Piezoelectric phase response and **g** piezoelectric amplitude response of phosphorene on the ITO/PET substrate

High-quality phosphorene is indispensable to the enhanced electrochemical properties of this nanocomposite MEA. Hence, we first investigated the properties of those exfoliated phosphorene nanosheets prior to conducting the subsequent assembly procedures (Fig. 1). Figure 1a illustrates the color variation of the exfoliated phosphorene in NMP solvent. With the prolonged exfoliation treatment, the solution gradually turned into blackish green, revealing the typical characteristics of solute phosphorene [48, 49]. Transmission electron microscopy (TEM) and HRTEM images shown in Figs. 1b and S1 clearly present the phosphorene with stacked folds, and the size of as-prepared phosphorene nanosheets ranged from 100 to 500 nm. The distinct lattice fringes observed from the HRTEM images further substantiate its high crystallinity, and two discernable fringes of 5.2 and 2.8 Å correspond to its (020) and (040) planes, respectively [19, 28]. This also indicates that the LPE process would not distort the origin crystallinity of bulk BP but destroy the interlayer van der Waals forces. The photoluminescence (PL) spectrum of the as-prepared phosphorene is illustrated in Fig. 1c, and three typical peaks are detected at ~ 410, 440 and 464 nm, which could be ascribed to the electronic transitions from the lowest unoccupied molecular orbital (LUMO) to the highest occupied molecular orbital (HOMO) and other orbitals below HOMO [50]. According to the previous work, the PL spectrum is closely related to the thickness of BP, and those emission peaks correspond to the few-layered BP nanosheets (less than 8 nm) and nanoscale phosphorene quantum dots (PQDs) [51]. Hence, it could be deduced that the as-prepared phosphorene is comprised of few-layered phosphorene nanosheets and PQDs. To further verify its structure, the atomic force microscopy (AFM) is conducted as shown in Fig. 1d. Those AFM images expressly reveal that those proposed phosphorene nanosheets own the thickness ranging from 2 to 4 nm (about 4–7 layers) and the lateral size ranging from 100 to 500 nm, which is in line with the results of TEM characterization. Besides the nanosheets, numerous PQDs with the diameters of ~ 20–40 nm are also found next to the nanosheets, which is in accordance with the results of PL spectrum. It is not surprising to detect the phosphorene nanosheets and PQDs simultaneously in the product since it is hard for LPE method to uniformly exfoliate the precursors, and PQDs could be easily obtained through the long-time exfoliation treatment (ranges from 3 [28] to 5 h [51]). Note that the emergence of PQDs is deemed to be beneficial for the improvement of electrochemical performances since they can readily embed in Ti_3_C_2_T_x_ framework and provide more exposed active sites for Li-ions, thus greatly enhancing the reaction kinetics [30]. We then hired the PFM to verify its intrinsic piezoelectricity, which had been predicted by previous literature [52]. Figure 1e shows the test area of PFM and its height profile is in line with that of AFM images, further suggesting the few-layer structure of proposed phosphorene. The piezoelectric phase response and amplitude response of as-prepared phosphorene are illustrated in Fig. 1f and g, respectively. The phase response presents the rectangular hysteresis loops, indicating that the polarization direction of the proposed phosphorene nanosheets could be switched by 180°. In addition, the amplitude response possesses the butterfly-shaped loops, further revealing its desirable piezoelectric characteristic, which originates from its highly directional properties and non-centrosymmetric lattice structure. Note that this favorable piezoelectric performance not only endows it with great potential in nanomechanical applications (such as nanogenerators) [33], but also improves its electrochemical kinetics by leveraging the self-built-in piezoelectric potential as an extra accelerator for alkali-ions [34].

**Fig. 2:**
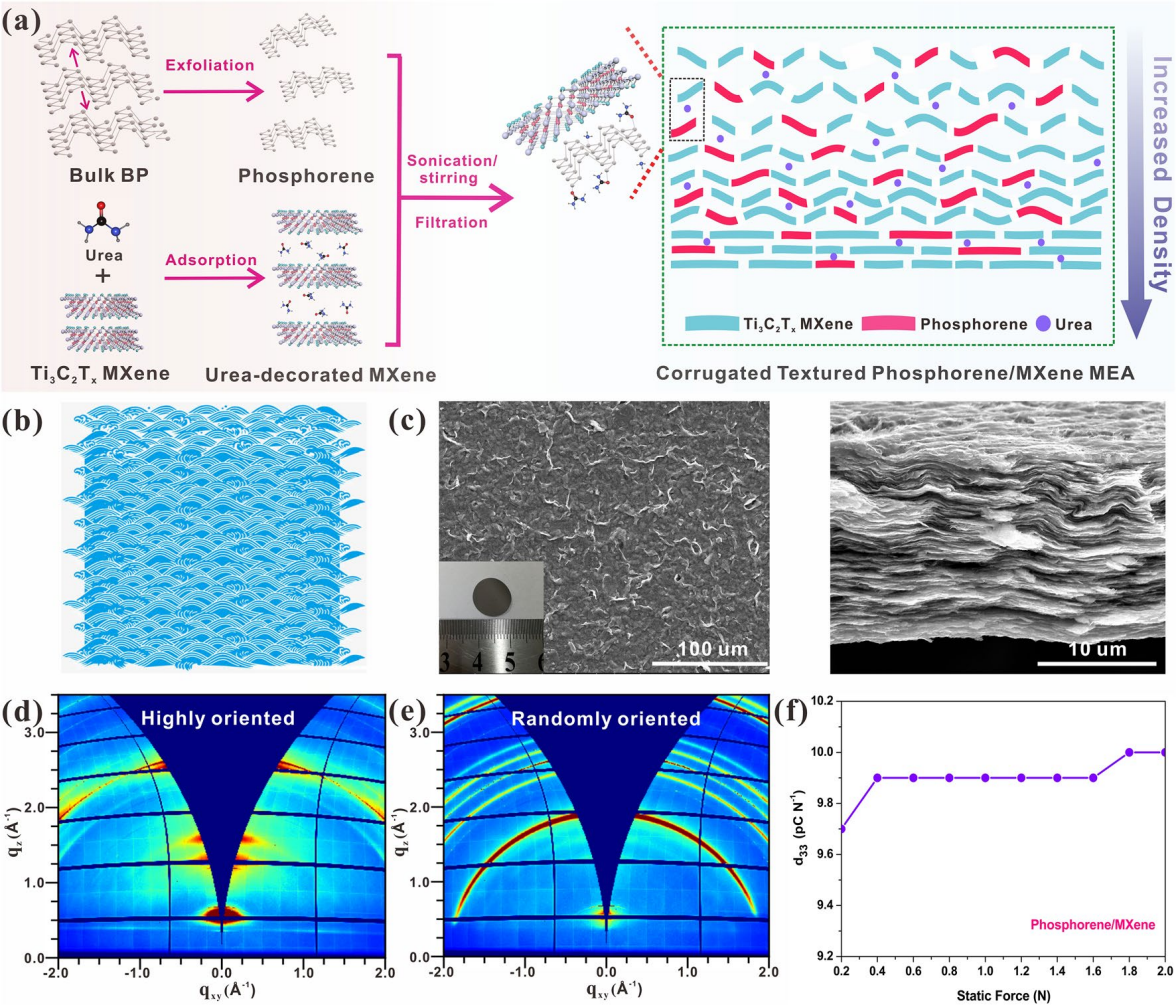
**a** Schematic illustration of the fabrication processes of the corrugated textured phosphorene/Ti_3_C_2_T_x_ nanopiezocomposite. **b** Typical corrugated patterns. **c** Surface and cross-sectional SEM images of phosphorene/Ti_3_C_2_T_x_ MEA. The typical GIWAXS images of **d** pure MXene MEA and **e** phosphorene/Ti_3_C_2_T_x_ MEA. **f** Quasistatic piezoelectric coefficient *d*_33_ of the proposed phosphorene/MXene MEA

**Fig. 3:**
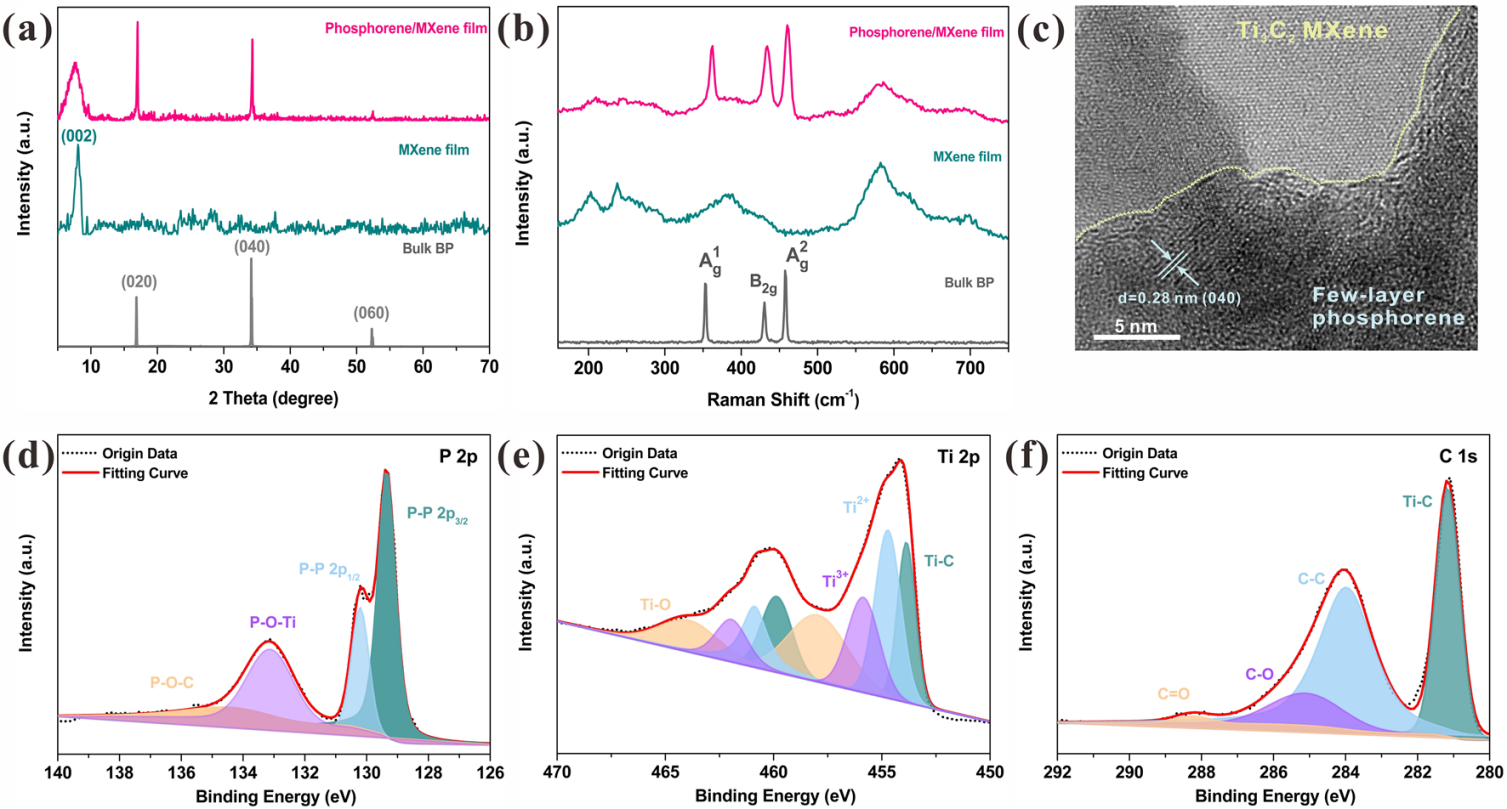
Characterization of as-prepared phosphorene/MXene MEA. **a** XRD spectra of these three materials and **b** their corresponding Raman spectra. **c** HRTEM image of the phosphorene/MXene MEA. The XPS spectra of the proposed phosphorene/MXene MEA nanocomposite in **d** P 2*p*, **e** Ti 2*p* and **f** C 1*s* region

Then, the properties of the proposed phosphorene/Ti_3_C_2_T_x_ MEA were detailedly characterized as displayed in Figs. 2 and 3. The overall synthesis procedures of the phosphorene/Ti_3_C_2_T_x_ heterostructure-textured MEA are illustrated in Fig. 2a. Both the phosphorene and few-layer Ti_3_C_2_T_x_ nanosheets were exfoliated from their bulk counterparts prior to the subsequent self-assembly process. According to the previous work, phosphorus is able to form the P–O–Ti covalent bonds with Ti_3_C_2_T_x_ MXene [30, 53], endowing it with the capability to assemble with Ti_3_C_2_T_x_ MXene. However, this assembly procedure is unable to modulate the tendency of assembly sites, in other words, a part of phosphorene could be anchored on the outer surface of Ti_3_C_2_T_x_ nanosheets rather than the interlayer. As a result, the volume variation and aggregation of those outer phosphorene are irrepressible without the Ti_3_C_2_T_x_ roof, leading to the inferior performance. Herein, the multifunctional polar urea molecules were exploited to assist the MEA structural regulation based on the following reasons: (1) Ti_3_C_2_T_x_ MXene owns the intrinsic capability to adsorb the urea inside its interlayer [54]; therefore, urea can arrive the interlayer of Ti_3_C_2_T_x_ readily through the simple mixing and stirring processes and contribute its carbonyl to form C–O–P covalent bonds to tightly bind the phosphorene inside in the interlayer of Ti_3_C_2_T_x_ through the stirring and ultrasonic treatments [19]; (2) it is suggested that urea could release the ammonium-ion to assist the homogenous dispersion of phosphorene in the interlayer of Ti_3_C_2_T_x_ MXene [26]; (3) the decomposition of partial urea molecules under stirring and ultrasonic treatments can release gases, resulting in the corrugated surface of the MEA and the tilted and vertical orientation of phosphorene and Ti_3_C_2_T_x_ nanosheets, which is beneficial to the increase of overall piezoelectricity of this nanopiezocomposite and the lithium-ion transfer. In other words, it is necessary for a polar urea additive to bridge or cross-link phosphorene and Ti_3_C_2_T_x_ MXene to form a more stable framework of an ionic or charged type 2D/2D layered heterostructure during electrostatic interaction/adsorption and assembly processes since both 2D phosphorene and Ti_3_C_2_T_x_ MXene generally possess the same negative electrostatic charges. In addition, beyond the traditional skin effect of the porous membrane formation, and bio-inspired by Murray membrane construction such as a leaf-vein with hierarchical porous networks and the corresponding model, it was illustrated that biomimetic Murray porous asymmetric membrane can be synchronously engineered using a polar urea as a multifunctional additive. Therefore, as shown in Fig. 2a, a scalable, stable and hierarchically porous asymmetric membrane electrode was assembled, not to mention the orientation and adjustment of the textured nanopiezocomposite during the above-mentioned membrane formation processes and control methods. By adopting the urea-assisted self-assembly process and subsequent filtration and freeze-drying procedures, the textured Ti_3_C_2_T_x_ MXene MEA with homogenous interbedded phosphorene was prepared and could be directly served as the anode of LIB. In addition, the typical corrugated structure (Fig. 2b) could be found on the surface of proposed phosphorene/Ti_3_C_2_T_x_ MEA (Fig. 2c), and those wrinkles are regarded as an advantage for the transfer of lithium ions [55]. Since the Ti_3_C_2_T_x_ is in the majority, the filtered phosphorene/MXene nanocomposite membrane could still retain its typical layered structure as illustrated in the cross-sectional SEM image. To further verify the influence of urea on the structure of the proposed nanocomposite, a contrast experiment was conducted and the corresponding EDS mapping images are displayed in Figs. S2 and S3. As a result, phosphorene nanosheets are more likely to be restacked or unevenly distributed in products through the direct self-assembly process without the urea. By comparison, the introduction of urea could greatly ameliorate these phenomena and those phosphorene nanosheets present a more uniform distribution in the nanocomposite MEA, which is considered to be a crucial factor of its electrochemistry properties. Note that the Ti_3_C_2_T_x_ MXene not only provides the highly conductive framework for phosphorene nanosheets to accommodate, but also improves the mechanical properties of BP. Figure S4 illustrates the load–displacement curves of these three samples and their calculated values of modulus and hardness. With the assistance of MXene framework, the modulus and hardness values of BP are improved to 4.115 and 0.053 GPa from the pristine 1.325 and 0.038 GPa, respectively, indicating the significant improvement of mechanical properties. It is noteworthy that the high modulus of nanoarchitecture is crucial for those active materials possessing severe volume expansion (such as P and Si), since the internal cracks could be effectively restrained and the structural stability is enhanced during the lithiation/delithiation processes, resulting in the enhanced long-term cycling performances [56]. To investigate the orientation distribution of these phosphorene nanosheets on the surface of Ti_3_C_2_T_x_, we further conducted the GIWAXS tests as illustrated in Figs. 2d, e and S5. The pure MXene film prepared through the filtration method owns a typical layered structure; hence, a strong reflection along the *q*_*z*_ direction could be detected, indicating the highly oriented arrangement of the 2D Ti_3_C_2_T_x_ nanosheets [57]. By contrast, the phosphorene/MXene MEA presents the rings with uniform intensities, suggesting that those phosphorene nanosheets existing on the Ti_3_C_2_T_x_ surface have no preference for the “flat” orientation of Ti_3_C_2_T_x_ and their arrangement is sort of random (which could be also seen in the SEM image that the numerous wrinkles are found on the surface of prepared nanocomposite). Compared to those orderly flat Ti_3_C_2_T_x_ nanosheets, this exoteric nanoarchitecture is able to significantly accelerate the electrolyte filtration and provide more channels for Li-ion transfer. Note that though the phosphorene nanosheets on the surface exhibit the comparatively random orientation, the proposed nanopiezocomposite still favorably inherits the piezoelectricity of phosphorene and presents the desirable *d*_33_ coefficients ranges from 9.7 to 10.0 pC N^−1^ as illustrated in Fig. 2f. Specifically, this satisfying piezoelectric response is deemed to possess positive influence on boosting the Li-ion kinetics by generating the extra piezoelectric potential serving as the extra accelerator for those Li-ions, and the possible mechanism will be discussed later.

To further analyze the structure and composition of the phosphorene/MXene MEA, more characterizations were conducted as shown in Fig. 3. Figure 3a illustrates the XRD spectra of bulk BP, Ti_3_C_2_T_x_ and the as-prepared phosphorene/Ti_3_C_2_T_x_ membrane. Three dominant peaks of bulk BP are detected at 16.8°, 34.1° and 52.3° which correspond to its (020), (040) and (060) planes, respectively, indicating the high crystallinity and purity of the phosphorene precursor. The characteristic peak of Ti_3_C_2_T_x_ is observed at ~ 7.2° rather than ~ 9.5° (the characteristic peak of Ti_3_AlC_2_), suggesting the accomplished removal of Al layers through the mild etching strategy [58]. The prepared phosphorene/Ti_3_C_2_T_x_ nanocomposite owns both the characteristic peaks of bulk BP and Ti_3_C_2_T_x_, indicating the successful combination of these two materials. Furthermore, the (002) peak of Ti_3_C_2_T_x_ shifts to the lower angle in the spectrum of nanocomposite, revealing that the nanocomposite exhibits the larger interlayer distance after the self-assembly process, which is supposed to facilitate the Li-ion intercalation and transfer. Figure 3b illustrates the comparison of Raman spectra of these three materials. Similarly, almost all the characteristic peaks belong to bulk BP and Ti_3_C_2_T_x_ are reflected in the spectrum of as-prepared phosphorene/Ti_3_C_2_T_x_ nanocomposite, which further verifies the successful combination of BP and Ti_3_C_2_T_x_ MXene. Note that a slight blue-shift occurs on the characteristic peaks of phosphorene in the proposed nanocomposite, indicating the existence of few-layered phosphorene and PQDs [28, 29, 50]. The TEM and HRTEM images displayed in Figs. 3c and S6 further reveal the successful embeddedness of phosphorene nanosheets, which is deemed to possess high mechanical stability and bate the restacking of phosphorene. Besides the phosphorene nanosheets, numerous PQDs are also detected on the surface of MXene nanosheets shown in Fig. S7, which is in accordance with the AFM image illustrated in Fig. 1d. This fact indicates that not only the phosphorene nanosheets but also the PQDs could be fabricated through the sonication treatment. By virtue of their comparatively smaller size, PQDs are able to embed in the interlayer of MXene much easier via the self-assembly process and contribute extra capacity through the battery-capacitive dual-model energy storage (DMES) behaviors [30]. Moreover, the XPS tests were conducted to investigate the elemental composition and the corresponding chemical bonding of these three samples. Figure S8 demonstrates the P 2*p* spectrum of bulk BP and its characteristic doublet peaks are detected at 130.6 and 129.7 eV, corresponding to the 2*p*_1/2_ and 2*p*_3/2_, respectively. Besides, an extra peak is also observed at 134.4 eV, which is attributed to the oxidation of BP since it exhibits high tendency to be oxidized to form P_x_O_y_ [51]. Figure S9 shows the typical XPS spectra of Ti_3_C_2_T_x_ MXene. The peaks of Ti^2+^, Ti^3+^ and Ti–C bonds originate from its intrinsic structure while the Ti–O bonds derive from the connection to the outer oxygenic functional group and partial oxidation of Ti. Compared to that of bulk BP, two extra peaks are detected at 133.1 and 134.8 eV of phosphorene in the nanocomposite spectrum as shown in Fig. 3d. Based on the previous literatures, BP is able to form the P–O–Ti bonds with Ti_3_C_2_T_x_, and the peak located at 133.1 eV could be attributed to that rather than the formation of P_x_O_y_ [53, 59]. In addition, it could be deduced that the peak located at 134.8 eV is ascribed to the formation of P–O–C bonds due to the addition of urea. Figure 3e and 3f demonstrate the Ti 2*p* and C 1*s* spectra of Ti_3_C_2_T_x_ in the nanocomposite, respectively. Note the Ti–O bonds of the Ti_3_C_2_T_x_ in the nanocomposite possess a larger area ratio (~ 25.8%) compared to that of pure Ti_3_C_2_T_x_ (~ 16.5%), which may be caused by the extra formation of Ti–O–P bonds and the further oxidation during the composite fabrication process, and its slight variation suggests the electron transfer process between the phosphorene and Ti_3_C_2_T_x_ [30]. In addition, an extra C=O bond is observed at C 1*s* in the nanocomposite spectrum, which is derived from the urea. Figure S10 displays the full survey XPS spectrum of the as-prepared phosphorene/Ti_3_C_2_T_x_ nanocomposite, and all the typical elements of phosphorene, Ti_3_C_2_T_x_ and urea are detected, suggesting the successful combination of these three materials. By analyzing the XPS results, the mechanism of urea-assisted self-assembly processes could be deduced as follows: first, the Ti_3_C_2_T_x_ plays the role of conductive frame, and the phosphorene could be assembled in the surface of Ti_3_C_2_T_x_ by forming the Ti–O–P bonds. However, this assembly process has no selectivity and phosphorene could be anchored on either the outer Ti layer or the internal Ti layer. Once plenty of the phosphorenes encounter on the outer surface, they may pile up again and the volume variation cannot be suppressed without the extra Ti_3_C_2_T_x_ roof. Here, a small quantity of urea was introduced to boost the uniform distribution of phosphorene and increase their tendency of being anchored in the interlayer on few-layered Ti_3_C_2_T_x_ MXene by releasing ammonium-ion and in-situ forming C–O–P bonds [26].

### Electrochemical Performances

**Fig. 4:**
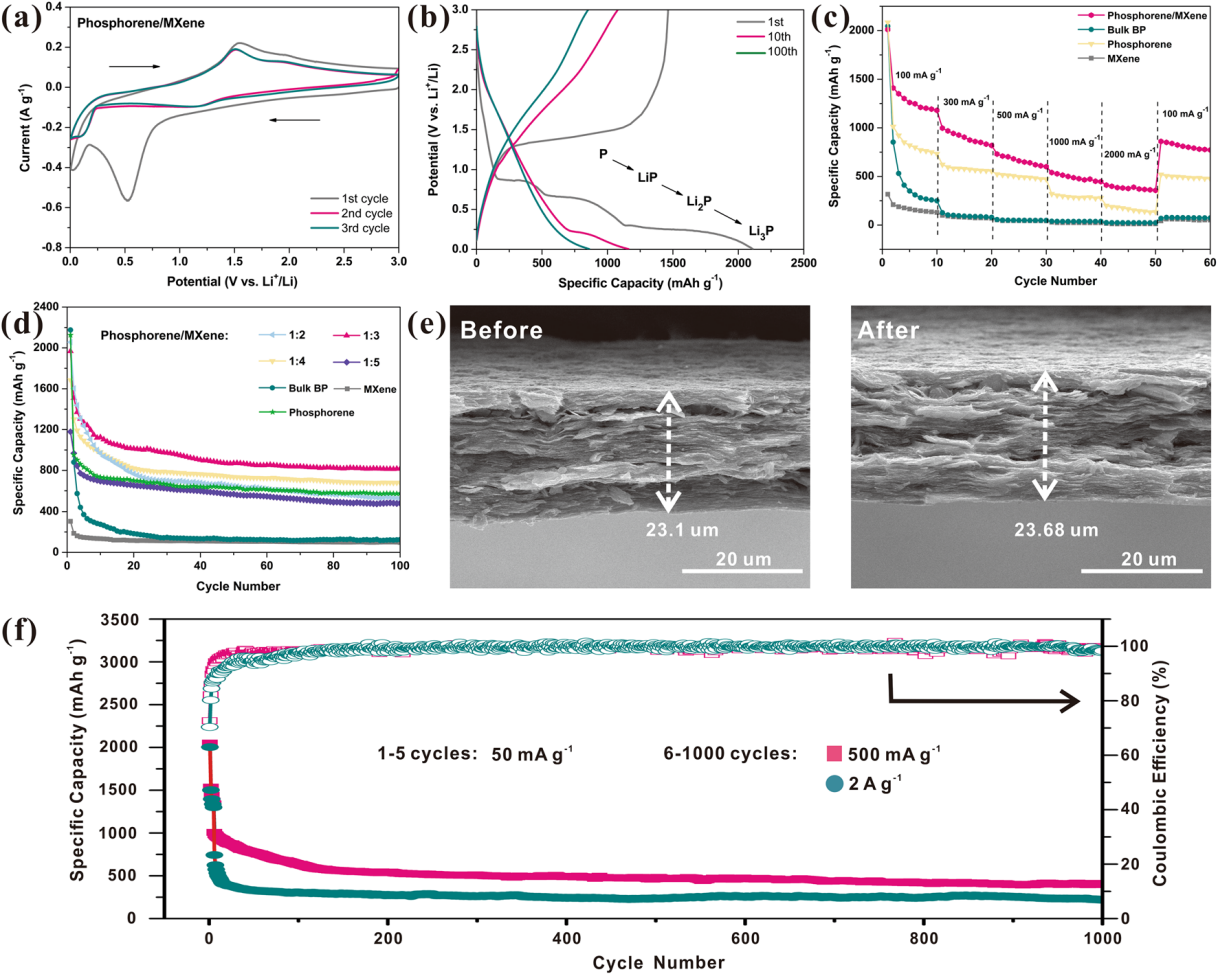
Electrochemical performance of the self-assembled phosphorene/Ti_3_C_2_T_x_ MEA. **a** The first three CV curves of phosphorene/Ti_3_C_2_T_x_ MEA (1:3) at 0.5 mV s^−1^ and **b** its first, tenth and hundredth charge/discharge curves at 100 mA g^−1^. **c** Rate performances of bulk BP, phosphorene, Ti_3_C_2_T_x_ and phosphorene/Ti_3_C_2_T_x_ MEA. **d** Cycling performances of bulk BP, phosphorene, Ti_3_C_2_T_x_ and various ratios phosphorene/Ti_3_C_2_T_x_ MEAs at 100 mA g^−1^. **e** Comparison of cross-sectional SEM images of the initial state and cycled state (100 cycles at 100 mA g^−1^). **f** Long-term cycling stability of phosphorene/MXene MEA and the corresponding coulombic efficiencies (1–5 cycles: 50 mA g^−1^ and 6–1000 cycles: 500 mA g^−1^ or 2 A g^−1^)

To evaluate the electrochemistry properties of as-prepared textured phosphorene/Ti_3_C_2_T_x_ MEA, the half-cells with lithium metal counter electrodes were assembled and their performances are displayed in Fig. 4. Figures 4a and S11, S12, S13 demonstrate the first three CV curves of these four electrodes, respectively. There are five dominant reduction peaks (1.68 V: P → LiP_7_; 1.35 V: LiP_7_ → LiP_5_; 0.69 V: LiP_5_ → LiP; 0.47 V: LiP → Li_2_P; 0.1 V: Li_2_P → Li_3_P) and one oxidation peak (~ 1.3 V: Li_*x*_P → Li_*y*_P, 1 ≤ *y* < *x* ≤ 3) are detected in CV curves of bulk BP and phosphorene electrode, indicating the stepwise lithiation alloying and delithiation dealloying processes of BP [26, 30, 38]. In addition, the reduction peaks (~ 0.7, 1.1 V) and oxidation peaks (~ 1.5, 2.2 V) emerged in that of pure Ti_3_C_2_T_x_ electrode also verify its capability to accommodate Li-ions (Ti_3_C_2_ + 2Li ↔ Ti_3_C_2_Li_2_) [60], albeit the capacity is comparatively low. As for the proposed phosphorene/Ti_3_C_2_T_x_ MEA, only four main redox peaks located at ~ 0.5, 0.1, 1.5 and 2.2 V are observed in the first cycle, and the corresponding process of each peak is similar to that of bulk BP electrode. However, the reduction peak at ~ 0.5 V is irreversible, which may be attributed to the formation of solid electrolyte interface (SEI) and the irreversible electrolyte reaction [26]. Except for the first cycle, the subsequent cycles are almost overlapped, suggesting the high reversibility of these three electrodes. Figures 4b, and S14, S15, S16 illustrate the first, tenth and hundredth charge/discharge curves of these four electrodes. Though the theoretical capacity of Ti_3_C_2_T_x_ MXene is up to 320 mAh g^−1^, its applicable charge capacity is only 161.9 mAh g^−1^, which is far from its upper limit [61], and only a reversible capacity of 81.7 mAh g^−1^ could be remained after 100 cycles at 100 mA g^−1^, therefore, Ti_3_C_2_T_x_ was hired to serve as the conductive framework rather than the main force of capacity contribution. Both the bulk BP, phosphorene and phosphorene/MXene MEA exhibit the superhigh initial discharge capacities (2110 mAh g^−1^ for bulk BP, 2125.6 mAh g^−1^ for pure phosphorene electrode and 2067.2 mAh g^−1^ for phosphorene/MXene MEA), whereas their coulombic efficiencies (CE) of the first cycle vary a lot. The bulk BP electrode presents a low reversible capacity of 671.1 mAh g^−1^ in the first cycle with 31.8% CE, and a low capacity of 102.7 mAh g^−1^ is remained after 100 cycles at 100 mA g^−1^, suggesting its inferior cycling stability, which could be ascribed to its unrestrained volume expansion. By virtue of the increased number of active sites, the phosphorene electrode retains a reversible capacity of 898.4 mAh g^−1^, which is larger than that of bulk BP electrode. However, it still suffers from the restacking and agglomeration of the phosphorene nanosheets, resulting in the unsatisfied cycling stability with the inferior capacity of 584.9 mAh g^−1^ after 100 cycles. By comparison, the phosphorene/Ti_3_C_2_T_x_ MEA exhibited the enhanced charge capacity of 1463.2 mAh g^−1^ in the first cycle with 70.8% CE, and the irreversible capacity could be attributed to the unidirectional formation of SEI and Li_3_P alloy (which would not transfer back to BP) [62]. Specifically, there are three distinct discharge plateaus observed in the first discharge curve, corresponding to the stepwise lithiation processes of phosphorene, and the detailed mechanism will be discussed later. Moreover, a favorable reversible capacity of 848.3 mAh g^−1^ could be remained after 100 cycles, indicating the enhanced cycling stability. The rate performances of these four electrodes are displayed in Fig. 4c. The specific capacity of bulk BP electrode suffers fast decay in the first tenth cycles and subsequently demonstrates the similar rate performances with that of MXene electrode. In contrast, pure phosphorene electrode and the proposed phosphorene/MXene MEA possess the desirable rate capability. Specifically, phosphorene/MXene MEA can retain the reversible capacities of 1410, 996, 720, 540 and 405 mAh g^−1^ at 100, 300, 500, 1000 and 2000 mA g^−1^, respectively. When the current density backs to 100 mA g^−1^, a high specific capacity of 878 mAh g^−1^ could be returned, suggesting the favorable rate capability. Subsequently, the influence of phosphorene ratio on the electrochemical properties was further studied as shown in Fig. 4d. Since the phosphorene plays the major role in contributing capacity, it is conspicuous that the initial capacities of these electrodes are proportionate to the BP contents. However, the excess phosphorene nanosheets may hard to find ample rooms for their accommodation and they may restack or agglomerate again, thus resulting in the fast decay in the preliminary stage (such as pure phosphorene electrode). Moreover, the agglomeration of superabundant phosphorene may also destruct the structural stability of proposed membrane electrode. Once the weight of phosphorene surpasses that of MXene (phosphorene/MXene > 1:1), the as-prepared MEA is much crispier and it is hard to obtain a complete free-standing membrane. On the contrary, when MXene occupies the predominant position, the nanocomposite MEA exhibits the comparatively steady cyclic performances, which is similar to that of pure MXene electrode. To balance the capacity and cyclic stability, the nanocomposite MEA comprised of phosphorene and MXene at a ratio of 1:3 was selected as the optimal ratio to be evaluated in other electrochemical characterizations and tests. To further verify the contribution of MXene on suppressing volume variation, the cross-sectional images were recorded at its initial and cycled states as illustrated in Fig. 4e. Only a negligible expansion (~ 2.5%) from 23.1 to 23.68 μm was observed after 100 cycles, indicating the desirable structural stability of the proposed MEA [18]. By comparison, both the bulk BP electrode (from 43.2 to 54.7 μm) and phosphorene electrode (from 14.1 to 16.7 μm) present the distinct volume variation after 100 cycles by combining with typical acetylene black with the volume expansion rates of 26.6 and 18.4%, respectively, as demonstrated in Fig. S17, which is responsible for the fast capacity decay. Figure 4f presents the long-term cycling stability of the proposed MEA. In general, severe electrode activation would occur when the sizes of active materials decrease to the nanoscale [30, 63, 64], which is known as a disadvantage for its practical applications. Herein, a proactive activation at low current density was conducted in the first five cycles to form the stable SEI, thus fully activating the electrode and avoiding the subsequent activation process. A superior reversible capacity of 1025 mAh g^−1^ could be achieved on the sixth cycle (the first cycle cycling at 500 mA g^−1^), and it remains the capacity of 826.7 mAh g^−1^ on the 33th cycle (~ 80% capacity retention). After 1,000 cycles at 500 mA g^−1^, a steady capacity of 420 mAh g^−1^ could be retained, corresponding to a negligible capacity loss of 0.059% per cycle. Moreover, a desirable capacity of 748, 574 and 237 mAh g^−1^ could be delivered on the sixth cycle, ninth cycle (~ 80 capacity retention) and 1,000 cycles at a high current density of 2 A g^−1^, respectively, suggesting the enhanced cycling stability (0.069% per cycle capacity loss). Though the optimal phosphorene/MXene electrode suffers comparatively fast capacity decay in the earlier stage, it presents steady cycling performances after the 80th cycle, suggesting its enhanced cycling stability [37, 38, 65]. In addition, all the CEs of subsequent cycles exceed 95% after 15 cycles, revealing its high reversibility and stable electrode structure. The enhanced electrochemical performances could be ascribed to the following reasons: (1) compared to the unexfoliated bulk BP, the phosphorene nanosheets possess more active sites for Li-ions to insert into/extract out the phosphorene in the omnidirectional way; (2) the outer MXene frame provides numerous ionic accesses for the transfer of Li-ions and enhances the overall conductivity which also accelerates the electron transfer; (3) the existence of PQDs could further accelerate the kinetics by virtue of their tremendous active sites and the synergistic effect with MXene [30]; (4) the corrugated textured surface further facilitates the transfer of lithium ions.

**Fig. 5:**
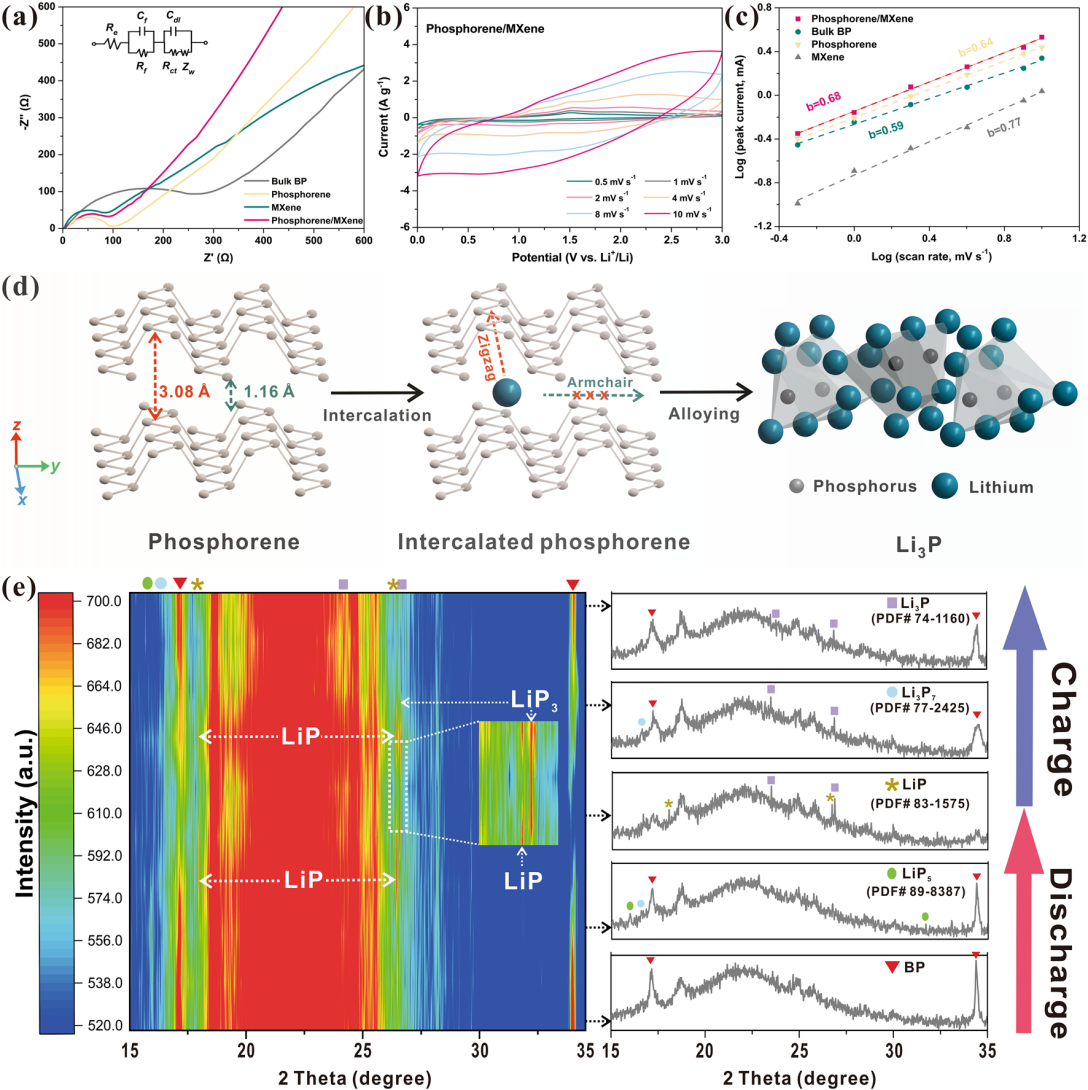
The electrochemical kinetics of self-assembled phosphorene/Ti_3_C_2_T_x_ MEA. **a** EIS spectra of these four electrodes and the corresponding equivalent circuit. **b** CV curves of phosphorene/MXene MEA at various scan rates. **c** Calculated *b* values of those four electrodes. **d** Reaction mechanism of phosphorene and **e** in situ XRD patterns of the synthesized phosphorene/MXene MEA

To further reveal the energy storage mechanism of the proposed phosphorene/MXene MEA, the EIS tests were conducted to compare the resistances of these four electrodes (Fig. 5a). Both the EIS spectra of these four electrodes are comprised of a semicircle in high-frequency region (corresponds to the SEI layer resistance *R*_*f*_ and the charge transfer resistance *R*_ct_) and an inclined line located in low-frequency region (relates to the Warburg impedance) [66]. By introducing the EIS values into the equivalent circuit model, these resistances could be calculated and the detailed fitted parameters are listed in Table S1, and the *R*_*e*_, which represents the internal resistance of a battery, was also included. Because of its semiconducting characteristics, the bulk BP electrode presents the highest values in all of the three kinds of resistances among these four electrodes, and its *R*_ct_ is as high as 267.4 Ω, which may be responsible for its inferior rate performances. In contrast, pure Ti_3_C_2_T_x_ electrode possesses desirable conductivity, resulting in the lowest resistances and favorable rate performances. Note that both the *R*_*f*_ and *R*_ct_ of the proposed phosphorene are dramatically decreased (from 56.47 to 30.23 Ω and 267.4 to 104.32 Ω, respectively) after exfoliation, and these values further decrease to 24.53 and 98.86 Ω, respectively, by rationally combining with MXene framework, suggesting the enhanced electron transfer capability during the lithiation/delithiation processes. The result strongly indicates MXene framework could effectively improve the poor conductivity of phosphorus, which is also in agreement with the enhanced electrochemical properties mentioned above. We then evaluated the *b* values of these three electrodes through the power-law relationship:

2 $$ i = {\text{ a}}v^{{\text{b}}} $$

whereas the *i* is the corresponding peak current in specific scan rate, *v* is the specific scan rate, *a* is the coefficient and *b* is the parameter indicating the different control mechanisms, which ranges from 0.5 to 1, corresponding to the capacitive-controlled process and diffusion-controlled process, respectively. Figures 5b and S18, S19, S20 depict the various CV curves of the phosphorene/Ti_3_C_2_T_x_ MEA, bulk BP, phosphorene and Ti_3_C_2_T_x_ electrodes, respectively. Based on the CV curves at various scan rates, the calculated *b* values of phosphorene/Ti_3_C_2_T_x_ nanocomposite MEA, bulk BP, phosphorene and pure Ti_3_C_2_T_x_ were 0.68, 0.59, 0.64 and 0.77, respectively (Fig. 5c). Both the electrodes exhibit two types of capacitive contributions, albeit with the differences of predomination contribution. Since bulk BP owns the high crystallization and ordered structure, its surface cannot provide numerous active sites or vacancy sites to generate pseudocapacitance, thus the bulk BP electrode is dominated by the capacitive-controlled process and exhibits the typical battery’s behaviors. By contrast, the MXene membrane which is fabricated by the exfoliated few-layered Ti_3_C_2_T_x_ nanosheets shows high compatibility of these two energy storage mechanisms, which is in line with the previous work [61]. Similarly, the proposed nanocomposite MEA and phosphorene also exhibit the intermediate *b* values, indicating their DMES mechanism. The alloying reactions occurred on phosphorene and PQDs (battery’s behaviors) play the major role in providing capacity, and the surface redox reactions occurred on MXene framework, PQDs and the extra exposed active sites of phosphorene (pseudocapacitive behaviors) could further accelerate the kinetics of Li-ion and effectively improve the total capacity. We then adopted the following formula to further quantify the capacity contributions of these two mechanisms:

3 $$ i \, \left( V \right) = k_{{1}} v^{{{1}/{2}}} + k_{{2}} v $$

where *k*_1_*v*^1/2^ and *k*_2_*v* represent the detailed contributions of diffusion-controlled process and capacitive-controlled process, respectively. As illustrated in Fig. S21, by virtue of the existence of MXene framework and abundant PQDs, the impact of pseudocapacitive contribution (~ 39.4%) cannot be neglected even at a comparatively small scan rate of 1 mV s^−1^, but the diffusion-controlled process still takes charge of the overall capacity contribution. Note that there is a small part of capacitive contribution located at low potential beyond the CV curve, which may be caused by the alloying process and electrode activation, resulting in the severe current variation and thus causing the calculated errors. This phenomenon is also commonly found in MXene [30] and other nanomaterials [67, 68], and here we had deducted the area of outer part to evaluate the capacitive contribution accurately. With the increase of scan rate, the capacitive contribution gradually gets the upper hand, and a high capacitive ratio of ~ 73.2% could be obtained at the scan rate of 10 mV s^−1^. Generally, the high ratio of capacitive contribution could efficiently boost the total capacity and rate capability [26], which may contribute to its improved electrochemical properties. As a result, by virtue of the rational structure design and the introduction of highly conductive MXene framework and PQDs, the proposed phosphorene/Ti_3_C_2_T_x_ nanocomposite MEA possesses the comparable performances among those phosphors-based LIB anodes listed in Table S2. Inspired by its boosted kinetics and efficient DMES mechanism, the optimal electrode was further tested at severe temperatures to dig its potential in low-temperature energy storage devices, and the corresponding discharge/charge curves are illustrated in Fig. S22. All the cells were stored at the corresponding temperatures for 1 h to achieve the thermodynamic equilibrium and cycled at 50 mA g^−1^ for 2 cycles to activate them and form the stable SEI layer prior to the relevant electrochemical tests. As expected, the optimal MEA (phosphorene/MXene 1:3) possesses the high reversibility and delivers the desirable capacities of 795 and 524 mAh g^−1^ at 0 and − 20 ℃, respectively, presenting great potential in harsh environments. Besides, BP is expected to possess the increased interlayer distance and lower bandgap at high-temperature conditions [69, 70], which is in favor of its electrochemical properties at high-temperature condition. In addition, black phosphorus possesses the comparatively higher Curie temperature (*T*_c_) [71], suggesting it can maintain the stable piezoelectricity in high-temperature, and its intrinsic piezoelectricity can still contribute to its high-temperature performances. As a result, the proposed phosphorene/Ti_3_C_2_T_x_ MEA is expected to perform better electrochemical properties at high temperatures, and exhibits great potential in wide-temperature-range energy storage devices. Furthermore, the in situ XRD technique was adopted to reveal the fast intercalation and alloying mechanism of phosphorene (Fig. 5d) [31], and trace the intermediate products as illustrated in Figs. 5e and S23. It’s noteworthy that the characteristic peaks of several intermediate products (such as LiP_5_, LiP and Li_3_P) are detected during the discharge/charge processes, albeit some of them are not obvious due to their comparatively low intensities. Note that all the intermediate products prior to Li_3_P (including LiP_5_, Li_3_P_7_ and LiP) were observed in the discharge process and then disappeared in the subsequent charge process, indicating the high reversibility of these intermediate products. However, the peaks of the final product Li_3_P still remained after the charge process with the weaker intensity, since they have been detected in the initial discharge process. It is generally believed that the Li_3_P is hard to convert to the pristine phosphorus state due to its destructed crystal structure [26, 72], whereas other research suggests the reversibility of Li_3_P [73]. Since the Li_3_P peaks still remained after the charge process, we prefer to assume that a large proportion of Li_3_P was able to convert to BP whereas the residual Li_3_P were still remained, thus causing severe volume variation and entire structural cracking of electrode. Furthermore, the ex-situ XPS tests were conducted to further reveal its lithiation/delithiation processes as depicted in Fig. S24. Similar to that of Fig. 3d, the P 2*p* spectrum at the initial state could be divided into two parts: the *p–p* peaks (including 2*p*_1/2_ and 2*p*_3/2_ peaks) and P–O peak (including P–O–Ti, P–O–C and partial oxidized P_x_O_y_). When it is discharged to 0.01 V, the original *p*–*p* peaks (belongs to P^0^ state) are almost disappeared, and the P–O bonds shift to the higher binding energy, indicating the full lithiation of the phosphorene [65]. As it is charged to 3 V, the *p–p* peaks are redetected, suggesting the reversibility of lithiation/delithiation processes of phosphorene. However, the peak area of *p–p* peak is decreased compared to that of its initial state, indicating the existence of partial unconverted intermediates, which is in agreement with the results of in-situ XRD spectra. In addition, the P–O peaks still remain at the higher binding energy area, indicating the strong interactions between the phosphorene and MXene, which is expected to be beneficial to the Li-ion transfer [65]. Specifically, it is suggested that the formation of Li_3_P could be inhibited by setting up the cut-off voltage (≥ 0.78 V) to achieve the higher cycling stability [62]. Herein, we put up a rational strategy to study the positive influence of this unique 2D/2D phosphorene/MXene nanoarchitecture on the general capability of lithium-ion storage and the voltage range had not been optimized specially. Therefore, the phosphorene/Ti_3_C_2_T_x_ MEA is expected to possess the higher cycling stability once the voltage range is elaborately designed in the future study, albeit with the lower total capacity (due to the lack of capacity contribution of the reaction step from LiP to Li_3_P).

**Fig. 6:**
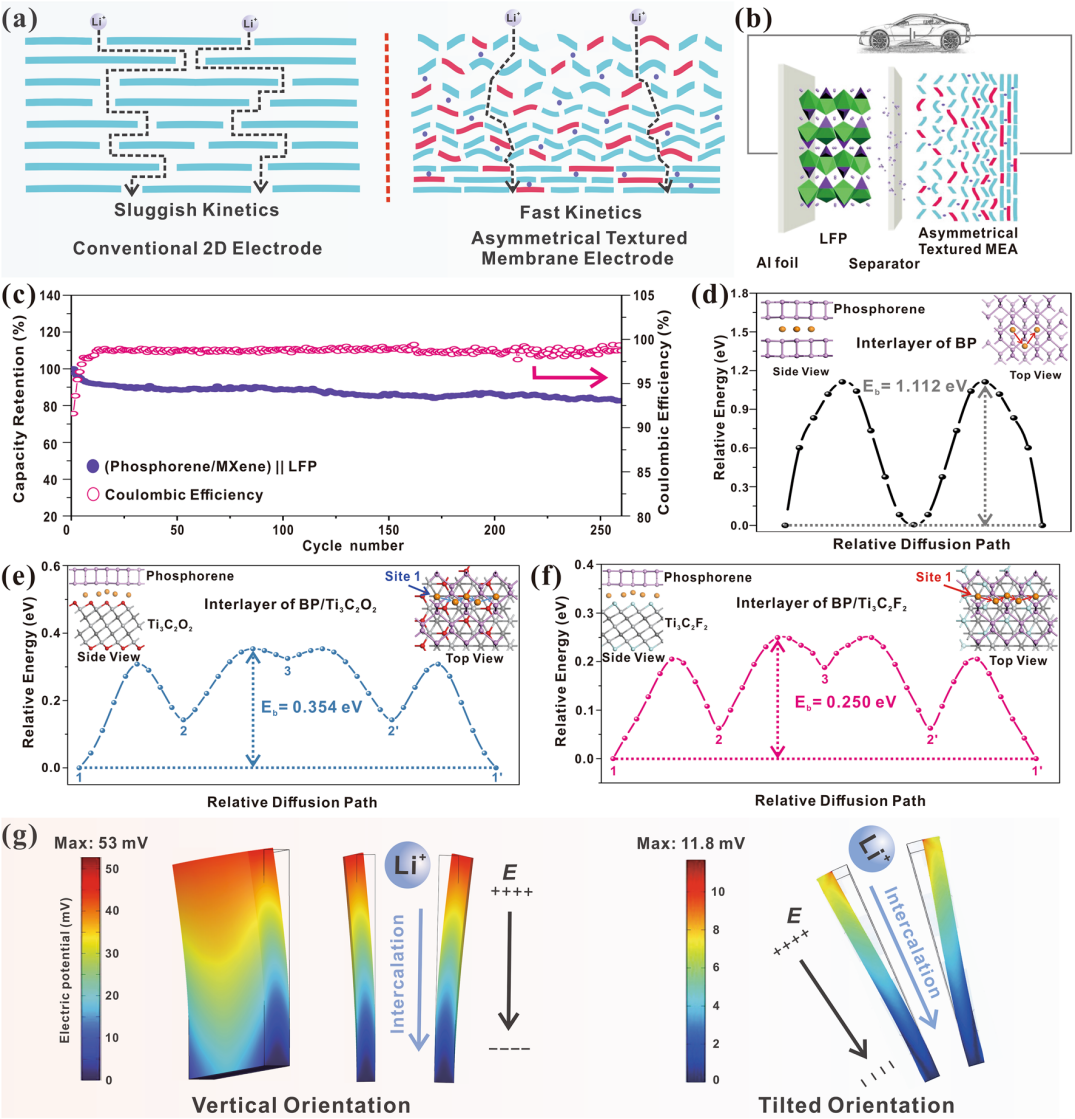
**a** Comparison of the lithium-ion transfer of conventional 2D electrode and the proposed asymmetrical corrugated textured MEA. **b** Schematic illustration of (phosphorene/Ti_3_C_2_T_x_ 1:3) || LFP full cell and its applications in power devices, and **c** its corresponding cycling performances at 0.2C (1C = 170 mAh g^−1^). All the full cells went through an activation procedure (charge/discharge at 0.05C for two cycles) prior to the evaluations. The diffusion paths from one stable site to another one and the calculated energy barriers of Li-ion diffusion on the interlayer of **d** bilayer BP, **e** phosphorene/Ti_3_C_2_O_2_ and **f** phosphorene/Ti_3_C_2_F_2_ heterostructures. All the calculated energies are relative to the energy of Li on its most stable adsorption site. **g** COMSOL simulation of corresponding piezoelectric potential generated during phosphorene alloying process

Figure 6a illustrates the comparison of the lithium-ion transfer of conventional 2D electrode and the proposed asymmetrical corrugated textured phosphorene/Ti_3_C_2_T_x_ MEA. The lithium-ions and electrolyte are hard to infiltrate into the conventional flat 2D electrode and it cannot provide sufficient channels for the transfer of lithium ions, resulting in the inferior transfer kinetics of lithium-ion. By contrast, the corrugated textured surface of the proposed phosphorene/Ti_3_C_2_T_x_ MEA, which is caused by the introduction of urea and the vacuum filtration, owns the open structure and it is able to provide numerous transfer channels for lithium-ions, thereby exhibiting the faster lithium-ion transfer kinetics. To further verify the applicability of the proposed phosphorene/Ti_3_C_2_T_x_ nanocomposite MEA, a typical commercial LIB cathode material—LFP, was adopted in the full-cell system tests as depicted in Fig. 6b. On the one hand, the proposed phosphorene/Ti_3_C_2_T_x_ MEA could be used as anode directly, thereby largely enhancing the energy density of energy storage device by waiving the mass of binder and current collector. On the other hand, its free-standing characteristics endow it with the great potentials in the futural flexible and wearable electrical devices. Figure 6c presents its long-term cycling performances, similar to that of the industrial procedures, an activation procedure is adopted prior to the subsequent tests to activate the system and obtain the stable SEI layer, and it exhibits a desirable capacity retention of 85.6% was obtained after 260 cycles at 0.2C and high CE, further indicating the high cycling stability of our proposed phosphorene/Ti_3_C_2_T_x_ MEA. Specifically, its energy density can be estimated using the following equation [74]:

4 $$ E = C_{{{\text{cathode}}}} \times m_{{{\text{cathode}}}} \times \left( {V_{{{\text{cathode}}}} - V_{{{\text{anode}}}} } \right)/(m_{{{\text{cathode}}}} + m_{{{\text{anode}}}} ) $$

where *E* represents energy density of cell, *C* represents capacity, *m* represents the mass of cathode or anode, *V* represents average working voltage of electrode materials. By introducing the relevant parameters, the energy density of this full cell at 0.2C is estimated at 365.5 Wh kg^−1^, which is comparable to other promising anode materials [74], and it is expected to possess the higher energy density by adopting other advanced cathode materials (such as NCM811).

To further study the advantages of this phosphorene/Ti_3_C_2_T_x_ nanopiezocomposite MEA theoretically and reveal the positive influence of the unique phosphorene/Ti_3_C_2_T_x_ heterostructure and its intrinsic piezoelectric effect on improving electrochemical kinetics, the relevant theoretical calculation and simulation were conducted. Here, the Ti_3_C_2_O_2_ and Ti_3_C_2_F_2_ were selected (–O and –F are the most common surface functional groups of MXene) to form the phosphorene/MXene heterostructure by stacking with phosphorene in the vertical direction through the interaction of van de Waals forces [32], and the adsorption and diffusion behaviors of Li-ion on Ti_3_C_2_T_x_, BP and their hybrid were revealed by the first-principles calculations. Figure S25 displays the adsorption energies of Li-ion in the interlayer of bilayer BP, BP/Ti_3_C_2_O_2_ and BP/Ti_3_C_2_F_2_, and the corresponding adsorption energies on monolayer Ti_3_C_2_O_2_ and Ti_3_C_2_F_2_ are also listed for comparison. As a result, it is suggested that the adsorption strength of Li-ion on Ti_3_C_2_T_2_ MXene will be enhanced with the incorporation of BP, indicating the boosted kinetics of the proposed nanocomposite. Specifically, the BP/Ti_3_C_2_O_2_ presents the strongest adsorption energy toward Li atom, revealing that the O-terminated phosphorene/MXene nanocomposite is more favorable for Li-ion storage. Furthermore, the relevant diffusion pathways and diffusion barriers of Li-ion in the interlayer of bilayer BP and BP/Ti_3_C_2_T_2_ heterostructures are investigated. As illustrated in Fig. 6d, there is only one adsorption site for Li-ion in the interlayer of BP with a high diffusion barrier of ~ 1.12 eV for Li-ion when moving from one site to another. As for the BP/Ti_3_C_2_T_2_ heterostructures, there are three suitable adsorption sites for Li-ion, and the most stable one is when Li on the hcp hollow site of C atom in Ti_3_C_2_T_2_, which is adopted in this theoretical calculation and denoted as site 1. The optimal diffusion pathway for Li-ion in the interlayer of BP/Ti_3_C_2_T_2_ is confirmed as moving from site 1 to an adjacent site 1’, following the pathway of 1 → 2 → 3 → 2’ → 1’ as indicated in Figs. 6e and 6f. Notably, the calculated diffusion barrier for Li-ion in BP/Ti_3_C_2_O_2_ and BP/Ti_3_C_2_F_2_ are 0.354 and 0.250 eV, respectively. It is noteworthy that the Li-ion diffusion barriers in BP/Ti_3_C_2_O_2_ and BP/Ti_3_C_2_F_2_ are significantly reduced compared to that in bilayer BP, revealing the greatly enhanced diffusion kinetics of Li-ions in BP/Ti_3_C_2_T_2_ nanostructures. To reveal the positive influence of its intrinsic piezoelectricity, the COMSOL software was adopted to simulate the self-built-in piezoelectric field generated during the discharge process as illustrated in Fig. 6g. Though the volume expansion of BP is greatly suppressed by the unique few-layer structure and the introduction of MXene framework, the slight volume variation is inevitable due to the formation of Li_3_P. As a result, the corresponding elastic strain will be exerted on these adjacent phosphorene nanosheets. Note that this generated elastic strain not only significantly increases the electromotive force of LIBs (which is substantiated in Li–Sn alloy system) [75], but also activates phosphorene to build the intrinsic piezoelectric potential along the direction of Li-ion diffusion, thereby effectively accelerating the Li-ion kinetics by providing the extra impetus. By introducing the parameters, the high intensities of the self-built-in piezoelectric field of ~ 0.13 mV Å^−1^ (vertical phosphorene nanosheets) and ~ 0.03 mV Å^−1^ (60° tilted phosphorene nanosheets) along the intercalation direction could be obtained to serve as the lithium-ion accelerator and improve its transfer kinetics. Based on the above simulation and calculations, the merits of the proposed textured phosphorene/Ti_3_C_2_T_x_ MEA could be summarized as follows: (1) this nanopiezocomposite favorably inherits the intrinsic piezoelectricity of phosphorene and the piezoelectric potential could be generated during the alloying process and served as the extra accelerator for Li-ions, thus accelerating the kinetics; (2) the synergistic effect between phosphorene and Ti_3_C_2_T_x_ could significantly lower the diffusion barriers of Li-ions, endowing it with the enhanced Li-ion kinetics; (3) the corrugated textured surface owns an open structure and it is able to provide more transfer channels for lithium ions; (4) the unique 2D phosphorene nanosheets and the existence of PQDs provide abundant active sites for Li-ions to storage and transfer, which effectively improves the total capacity of this MEA; (5) the introduction of highly conductive Ti_3_C_2_T_x_ framework could efficiently disperse those phosphorene nanosheets to avoid the aggregation and suppress the volume variation. Moreover, it further strengthens the structural stability by improving the overall modulus, thereby enhancing its rate and long-term cycling performances. Therefore, it is expected that those advantages mentioned above jointly contribute to the storage and diffusion behaviors of Li-ions, and the enhanced electrochemical properties were presented.

## Conclusions

In summary, a multifunctional polar urea-assisted synthesis strategy of scalable, stable and hierarchically porous asymmetric MEA of corrugated textured phosphorene/Ti_3_C_2_T_x_ MXene nanopiezocomposite was demonstrated. The phosphorene nanosheets and PQDs could be readily obtained through the LPE method and subsequently embedded in the MXene framework without apparent aggregation. By virtue of the presence of unique few-layered phosphorene and PQDs, abundant active sites were exposed and a battery-capacitive DMES mechanism was presented. Moreover, the intrinsic piezoelectricity of phosphorene was favorably inherited, thus providing the extra impetus for Li-ions by generating the piezoelectric potential and accelerating its kinetics. The introduction of outer Ti_3_C_2_T_x_ framework can not only suppress the volume variation of phosphorene and improve the structural stability by enhancing the modulus, but also lower the diffusion barriers of Li-ions through the synergistic effect between the phosphorene and Ti_3_C_2_T_x_, thereby significantly enhancing the kinetics of Li-ions. Benefiting from the effective combination of Ti_3_C_2_T_x_ framework and phosphorene and their unique advantages, the optimal phosphorene/Ti_3_C_2_T_x_ MEA exhibited improved cycling stability in both the half-cell and full-cell systems. A desirable capacity of 1463.2 mAh g^−1^ at 100 mA g^−1^ and the stable cycling performances (406.8 mAh g^−1^ at 500 mA g^−1^ after 1,000 cycles) could be delivered, indicating its great potential serving as the anode material of LIBs. Notably, it can deliver a highly reversible capacity of 524 mAh g^−1^ even at the low temperature of − 20 ℃, suggesting its desirable potential in harsh environment. Besides its intrinsic piezoelectricity, monolayer or few-layer phosphorene-based self-assembled nanocomposites might exhibit intrinsic pyroelectricity and ferroelectricity. In general, the polarization gradient fields increase with the decrease of temperature, which could further accelerate the electrochemical kinetics of the electrode, especially for the low-temperature and/or fast charging investigation. In addition, its stepwise reaction procedures were detailedly revealed by the in situ XRD technique and the partial irreversibility of Li_3_P was regarded as the chief culprit of the capacity loss mechanism. By regulating the potential range and further optimizing the overall structure (i.e., the crystallographically textured electrode), the value of phosphorene as active electrode material could be fully squeezed.

## Supplementary Information

Below is the link to the electronic supplementary material.

Supplementary file1 (PDF 2249 KB)
