# Supplementary material for: Textured Asymmetric Membrane Electrode Assemblies of Piezoelectric Phosphorene and Ti3C2Tx MXene Heterostructures for Enhanced Electrochemical Stability and Kinetics in LIBs
Source: Nanomicro Lett. 2024 Jan 8;16:79. doi: 10.1007/s40820-023-01265-5 (PMC10774488; doi:10.1007/s40820-023-01265-5)
Supplement: Supplementary file 1 — Supplementary file1 (PDF 2249 KB) [file 40820_2023_1265_MOESM1_ESM.pdf]

Nano-Micro Letters

Supporting Information for

Textured Asymmetric Membrane Electrode Assemblies of Piezoelectric Phosphorene and $Ti_3C_2T_x$ MXene Heterostructures for Enhanced Electrochemical Stability and Kinetics in LIBs

Yihui Li[1, 6, †], Juan Xie[2, †], Ruofei Wang[3], Shugang Min[3], Zewen Xu[1, 6, *], Yangjian Ding[1, 6], Pengcheng Su[1, 6], Xingmin Zhang[4], Liyu Wei[5], Jing-Feng Li[5], Zhaoqiang Chu[3], Jingyu Sun[1], and Cheng Huang[1, 6, 7, *]

[1]Volta and DiPole Materials Labs, College of Energy, Soochow Institute for Energy and Materials InnovationS (SIEMIS), Key Laboratory of Advanced Carbon Materials and Wearable Energy Technologies of Jiangsu Province, Key Laboratory of Core Technology of High Specific Energy Battery and Key Materials for Petroleum and Chemical Industry, Soochow University, Suzhou 215006, P. R. China

[2]School of Materials Science and Engineering, Jiangsu University, Zhenjiang 212013, P. R. China

[3]College of Underwater Acoustic Engineering, Harbin Engineering University, Harbin 150001, P. R. China

[4]Shanghai Synchrotron Radiation Facility, Shanghai Advanced Research Institute, Shanghai Institute of Applied Physics, Chinese Academy of Sciences, Shanghai 201204, P. R. China

[5]State Key Laboratory of New Ceramics and Fine Processing, School of Materials Science and Engineering, Tsinghua University, Beijing 100084, P. R. China

[6]High Density Materials Technology Center for Flexible Hybrid Electronics, Suzhou Institute of Electronic Functional Materials Technology, Suzhou Industrial Technology Research Institute, Suzhou 215151, P. R. China

[7]Institute of Advanced Materials and Institute of Membrane Science and Technology, Jiangsu National Synergistic Innovation Center for Advanced Materials, Suzhou Laboratory and Nanjing Tech University, Nanjing 211816, P. R. China

†Yihui Li and Juan Xie contributed equally to this work.

*Corresponding authors. E-mail: zwxu@suda.edu.cn (Zewen Xu); chengh@suda.edu.cn (Cheng Huang)

Supplementary Figures and Tables

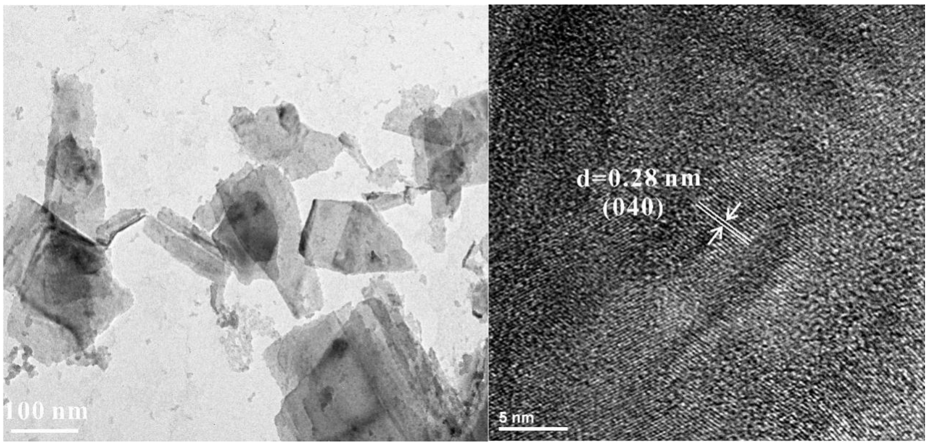

Fig. S1 TEM and HRTEM images of the as-exfoliated BP nanosheets

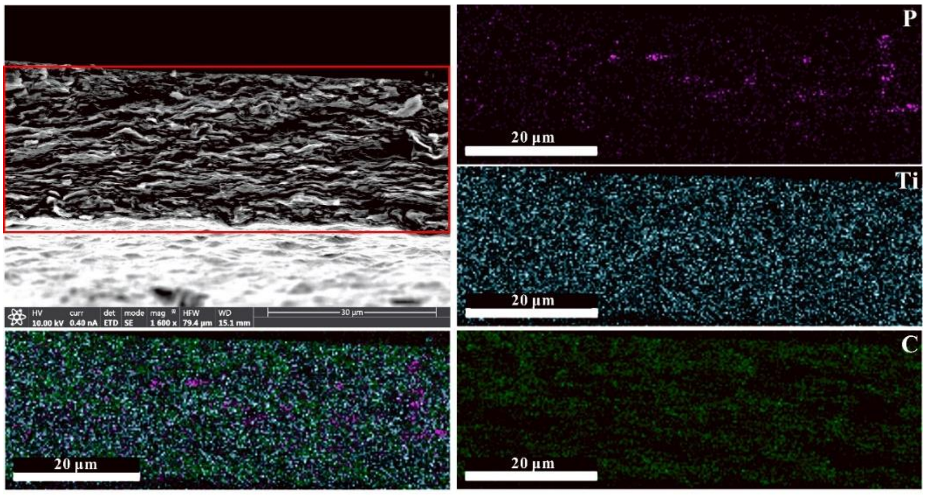

Fig. S2 The cross-sectional SEM image of the synthesized phosphorene/MXene MEA and its EDS mapping of P, Ti and C elements

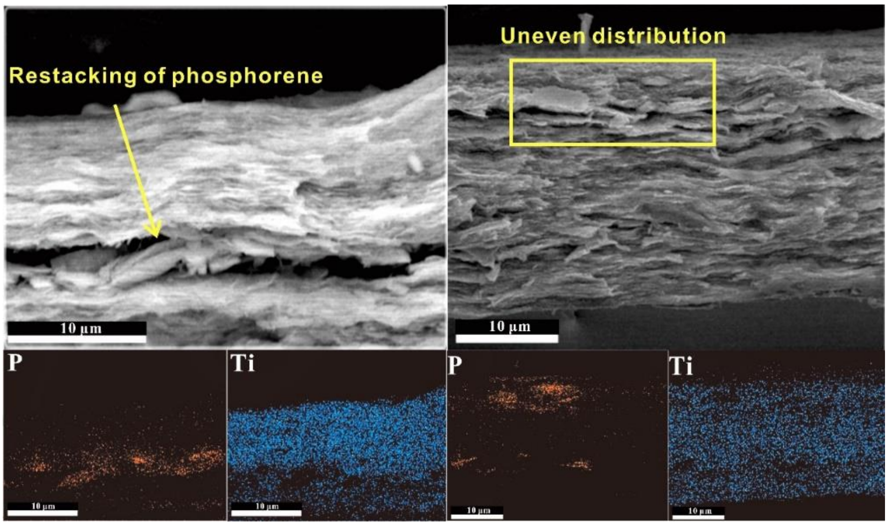

Fig. S3 The cross-sectional SEM image and EDS elemental mapping analysis of phosphorene/MXene MEA without urea assistance. The phenomena of the restacking and the uneven distribution of phosphorene nanosheets

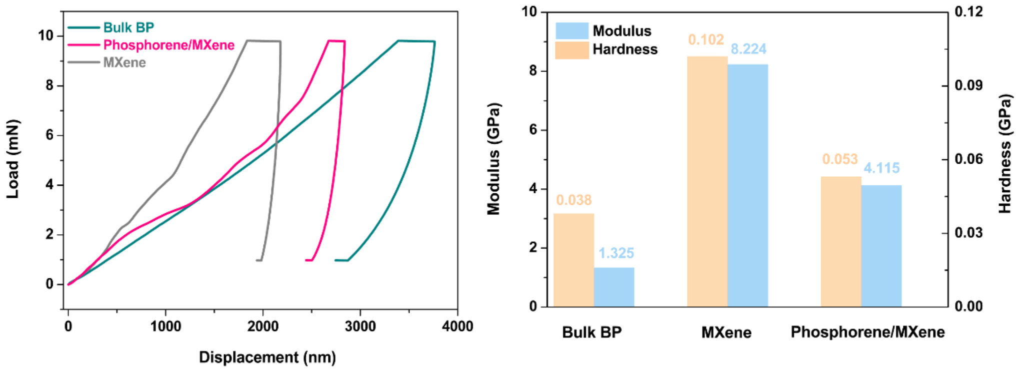

Fig. S4 The load-displacement curves of these three samples and the corresponding calculated values of modulus and hardness

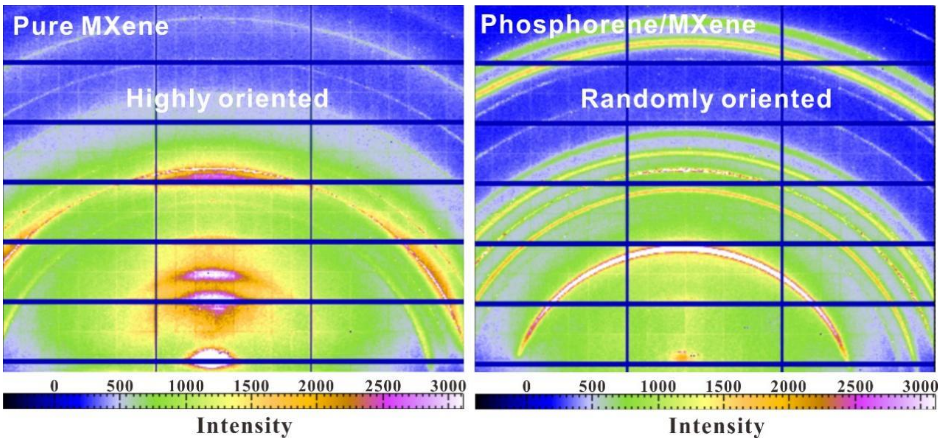

Fig. S5 The pristine GIWAXS profiles of pure $Ti_3C_2T_x$ MXene (left) and the proposed phosphorene/MXene MEA (right)

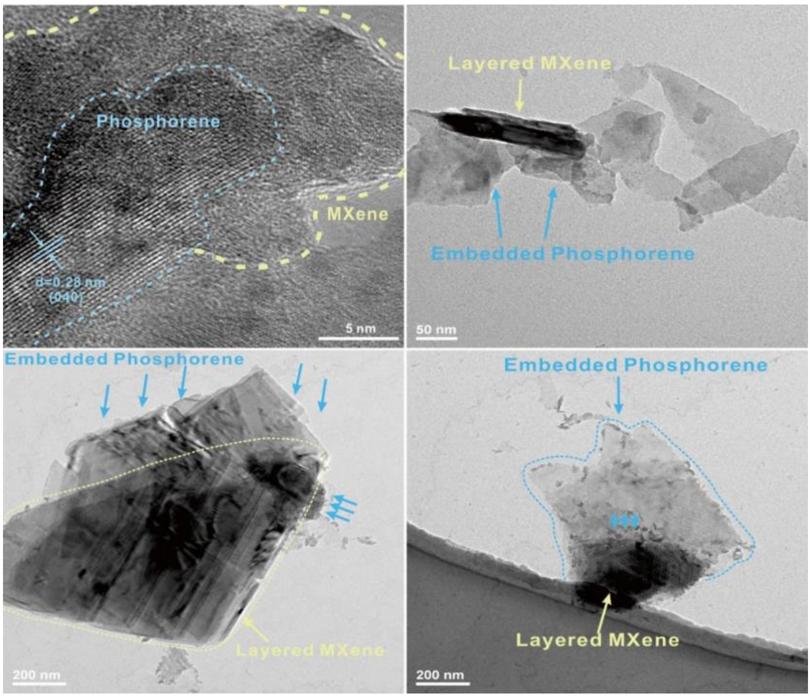

Fig. S6 The HRTEM and TEM images of the as-prepared phosphorene/MXene MEA

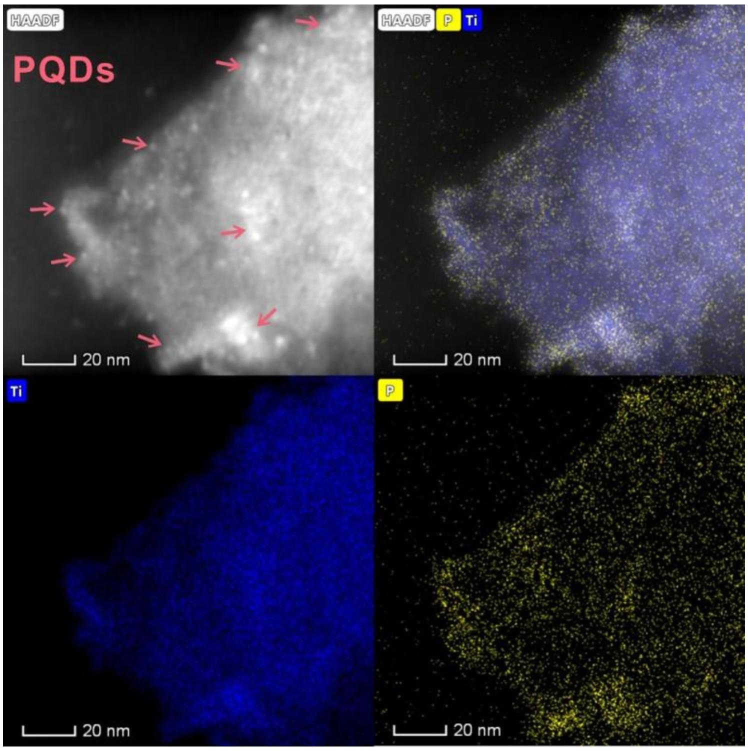

Fig. S7 The high-angle annular dark-field scanning transmission electron microscopy (HAADF-STEM) images of the synthesized nanocomposite and its EDS elemental mapping analysis of Ti and P elements. The light spots on the MXene surface correspond to the PQDs

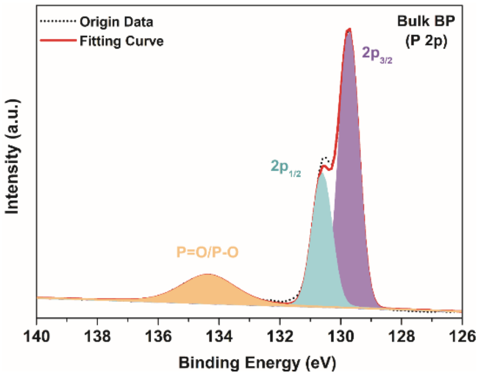

Fig. S8 The XPS spectrum of bulk BP in the P 2p region

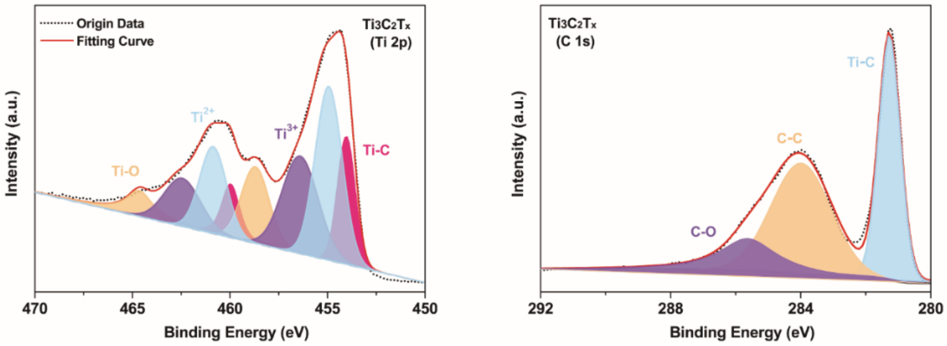

Fig. S9 The XPS spectra of pure $Ti_3C_2T_x$ MXene

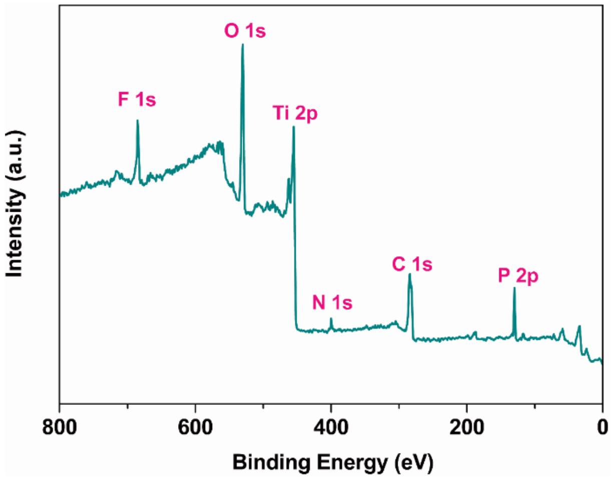

Fig. S10 The full survey XPS spectrum of proposed phosphorene/MXene MEA

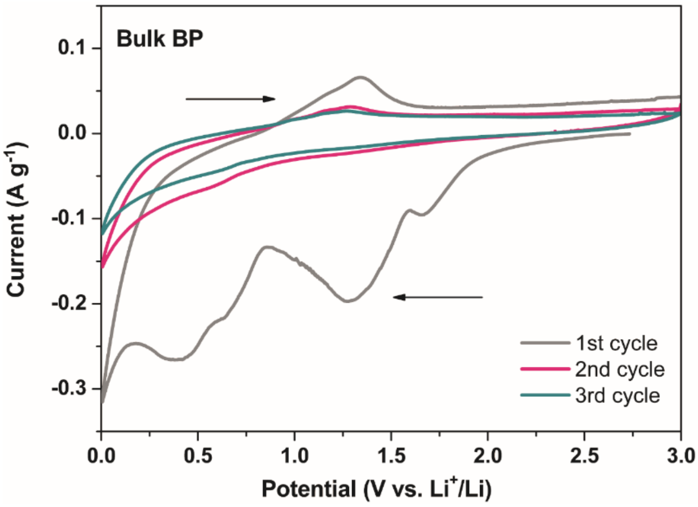

Fig. S11 The first three CV curves of bulk BP electrode at 0.5 mV s^{-1}

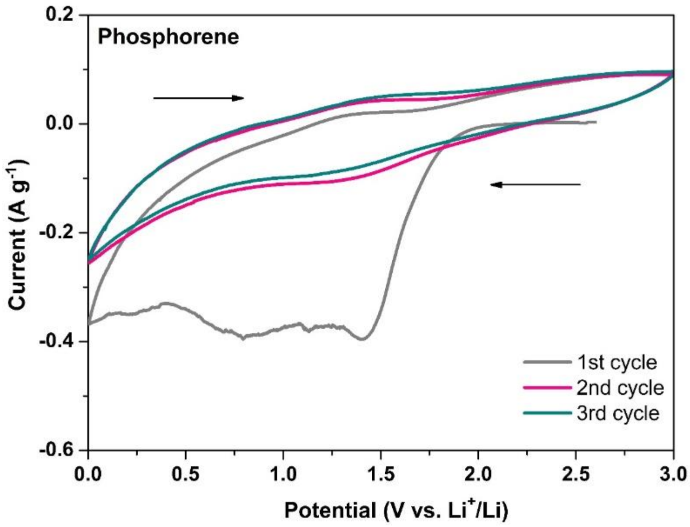

Fig. S12 The first three CV curves of phosphorene electrode at 0.5 mV s^{-1}

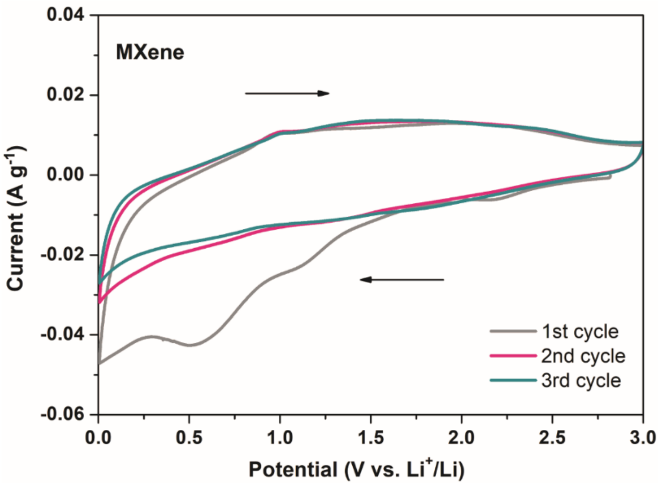

Fig. S13 The first three CV curves of $Ti_3C_2T_x$ MXene electrode at 0.5 mV s^{-1}

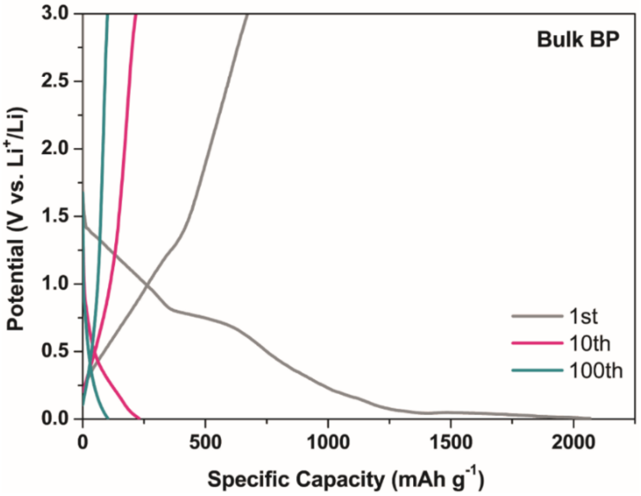

Fig. S14 The first, tenth and hundredth charge/discharge profiles of bulk BP electrode at 100 mA g^{-1}

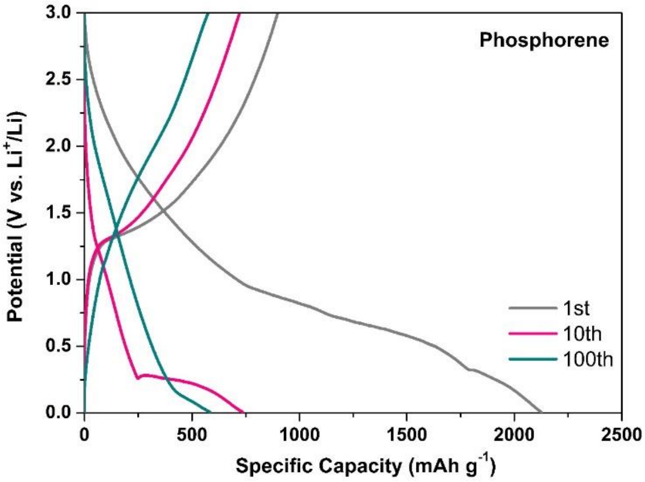

Fig. S15 The first, tenth and hundredth charge/discharge profiles of phosphorene electrode at 100 mA g^{-1}

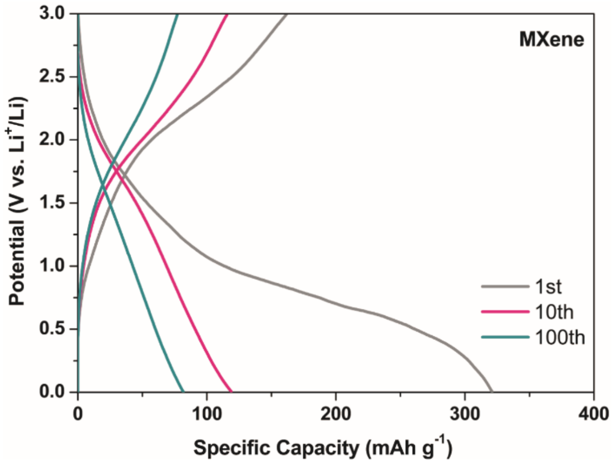

Fig. S16 The first, tenth and hundredth charge/discharge profiles of $Ti_3C_2T_x$ MXene electrode at 100 mA g

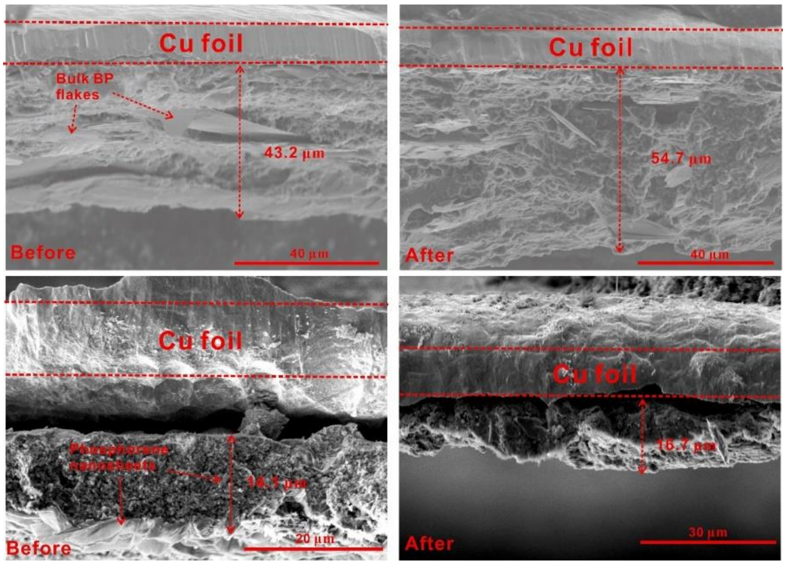

Fig. S17 The cross-sectional SEM images of bulk BP electrode (up) and phosphorene electrode (down) before and after 100 cycles at 100 mA g

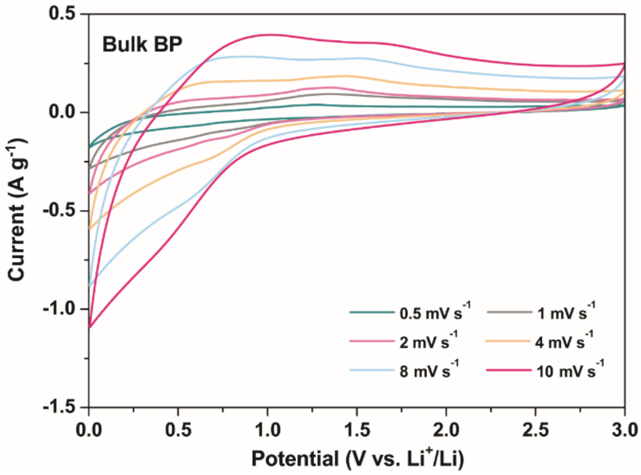

Fig. S18 The CV curves of bulk BP electrode at various scan rates

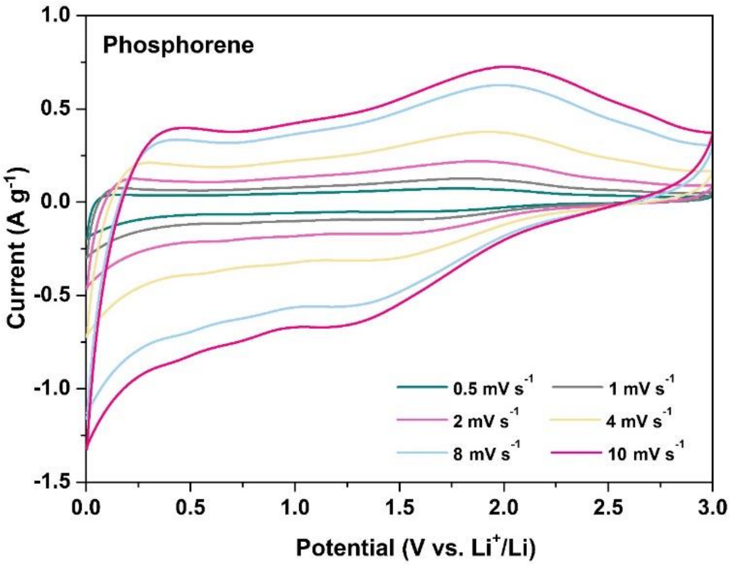

Fig. S19 The CV curves of phosphorene electrode at various scan rates

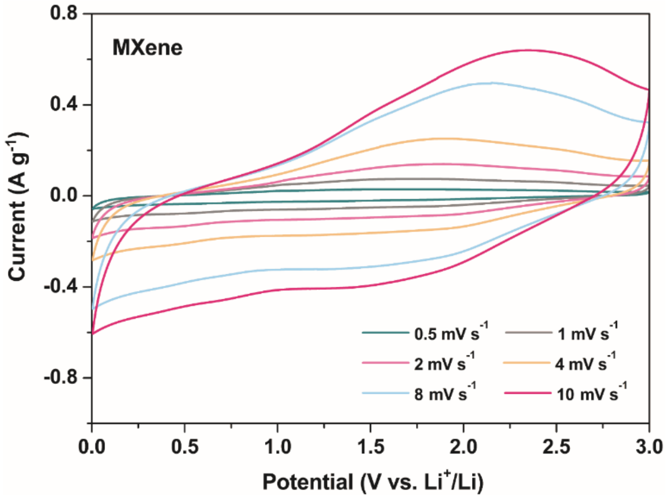

Fig. S20 The CV curves of $Ti_3C_2T_x$ MXene electrode at various scan rates

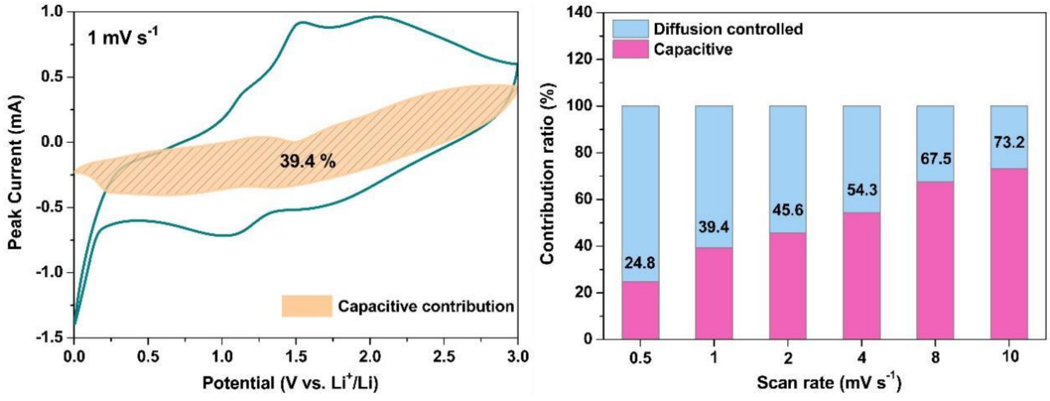

Fig. S21 The capacitive contribution of phosphorene/MXene MEA at the scan rate of 1 mV s^{-1}, and its corresponding capacitive contributions at various scan rates

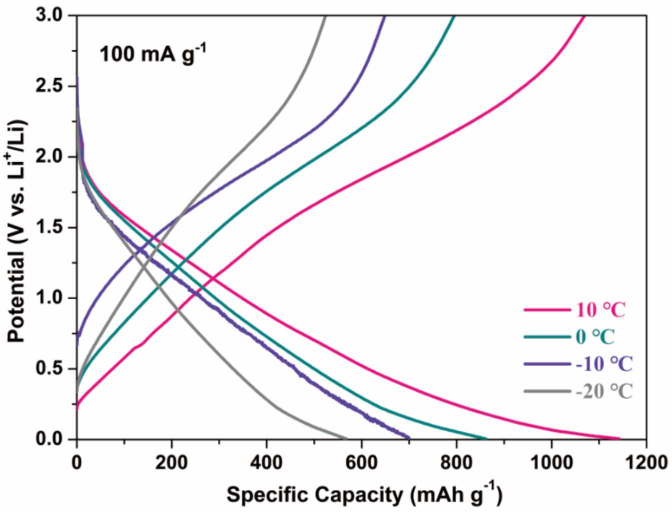

Fig. S22 The discharge/charge curves of the optimized electrodes (phosphorene/MXene 1:3) at various low-temperature conditions

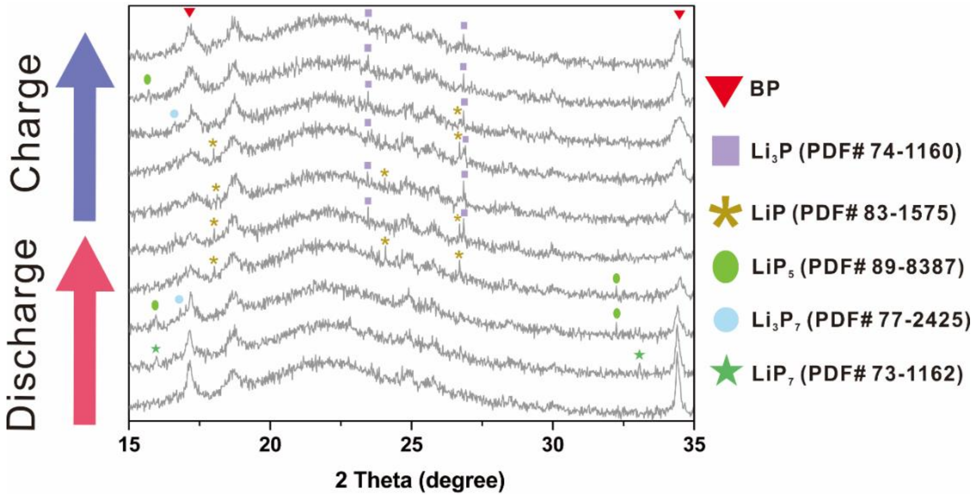

Fig. S23 The *in situ* XRD spectra of the synthesized phosphorene/MXene MEA

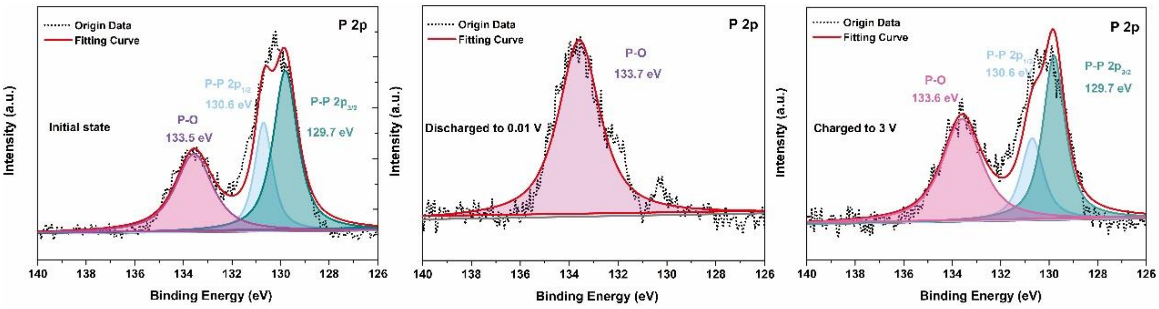

Fig. S24 The P 2p XPS spectra of phosphorene/MXene MEA at various states

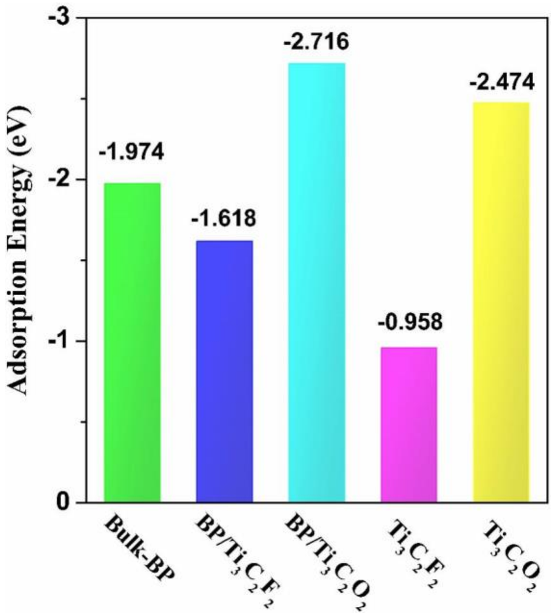

Fig. S25 The calculated adsorption energies of the most stable Li adsorption sites in these samples

Table S1 The corresponding impedance parameters of those four electrodes derived from the equivalent circuit model

**Electrode**	R_e **(Ω)**	R_f **(Ω)**	R_{ct} **(Ω)**
$Ti_3C_2T_x$ MXene	5.13	22.31	87.62
Bulk BP	6.48	56.47	267.4
Phosphorene	8.58	30.23	104.32
Phosphorene/MXene	5.91	24.53	98.86

Table S2 The electrochemical performances in comparison with other BP-based anode materials in LIBs

**Materials**	**Initial charge capacity (mAh g^{-1}) at (*X*) current density (mA g^{-1})**	**Reversible capacity (mAh g^{-1}) after (*Y*) cycles at (*Z*) current density (A g^{-1})**	**Methods**	**Refs.**
Phosphorene/$Ti_3C_2T_x$ nanocomposite	1463 (100)	406.8 (1000) (0.5); 230.2 (1000) (2)	LPE/filtration/ polar urea-assisted self-assembly	This work
BP quantum dots/ $Ti_3C_2T_x$ composite	~650 (100)	815 (700) (0.2); 520 (2400) (1)	LPE/liquid- solid-phase assembly	[S1]
BP-graphene hybrid paper	920 (100)	402 (500) (0.5)	LPE/filtration	[S2]

Phosphorus nanosheets	1969 (200)	1683 (100) (0.2)	Wet-chemical solvothermal	[S3]
Sandwiched BP/graphene hybrid[a)]	1836.8 (100)	1401 (200) (0.1)	Filtration	[S4]
(BP-graphite)/PANI	1650 (260)	440 (2000) (13)	Ball-milling	[S5]
BP nanoparticle-graphite composite[a)]	2270 (0.2 C)	1849 (100) (0.2C)	HEMM	[S6]
BP-carbon composite	1814 (100)	~600 (100) (0.1)	HEMM	[S7]
Phosphorus-graphene hybrid[a)]	2110 (260)	1283 (300) (0.26)	Ball-milling	[S8]
BP-carbon composite	1677 (100)	349 (50) (0.1)	HEMM	[S9]
BP-CNTs hybrid	2004 (100)	521.9 (650) (0.5)	Chemical cross-linking	[S10]

a) the capacity is calculated only based on the weight of BP

Supplementary References

[S1] R. Meng, J. Huang, Y. Feng, L. Zu, C. Peng, L. Zheng, L. Zheng, Z. Chen, G. Liu, B. Chen, Y. Mi, J. Yang. Black phosphorus quantum dot/Ti_3C_2 MXene nanosheet composites for efficient electrochemical lithium/sodium-ion storage. Adv. Energy Mater. **8**(26), 1801514 (2018). https://doi.org/10.1002/aenm.201801514

[S2] L. Chen, G. Zhou, Z. Liu, X. Ma, J. Chen, Z. Zhang, X. Ma, F. Li, H.-M. Cheng, W. Ren. Scalable clean exfoliation of high-quality few-layer black phosphorus for a flexible lithium ion battery. Adv. Mater. **28**(3), 510-517 (2016). https://doi.org/10.1002/adma.201503678

[S3] Y. Zhang, X. Rui, Y. Tang, Y. Liu, J. Wei, S. Chen, W. R. Leow, W. Li, Y. Liu, J. Deng, B. Ma, Q. Yan, X. Chen. Wet-chemical processing of phosphorus composite nanosheets for high-rate and high-capacity lithium-ion batteries. Adv. Energy Mater. **6**(10), 1502409 (2016). https://doi.org/10.1002/aenm.201502409

[S4] H. Liu, Y. Zou, L. Tao, Z. Ma, D. Liu, P. Zhou, H. Liu, S. Wang. Sandwiched thin-film anode of chemically bonded black phosphorus/graphene hybrid for lithium-ion battery. Small. **13**(33), 1700758 (2017). https://doi.org/10.1002/smll.201700758

[S5] H. Jin, S. Xin, C. Chuang, W. Li, H. Wang, J. Zhu, H. Xie, T. Zhang, Y. Wan, Z. Qi, W. Yan, Y.-R. Lu, T.-S. Chan, X. Wu, J. B. Goodenough, H. Ji, X. Duan.

Black phosphorus composites with engineered interfaces for high-rate high-capacity lithium storage. Science. **370**(6513), 192 (2020). https://doi.org/10.1126/science.aav5842

[S6] J. Sun, G. Zheng, H.-W. Lee, N. Liu, H. Wang, H. Yao, W. Yang, Y. Cui. Formation of stable phosphorus–carbon bond for enhanced performance in black phosphorus nanoparticle–graphite composite battery anodes. Nano Lett. **14**(8), 4573-4580 (2014). https://doi.org/10.1021/nl501617j

[S7] C. M. Park, H. J. Sohn. Black phosphorus and its composite for lithium rechargeable batteries. Adv. Mater. **19**(18), 2465-2468 (2007). https://doi.org/10.1002/adma.200602592

[S8] Z. Yu, J. Song, M. L. Gordin, R. Yi, D. Tang, D. Wang. Phosphorus-graphene nanosheet hybrids as lithium-ion anode with exceptional high-temperature cycling stability. Adv. Sci. **2**(1-2), 1400020 (2015). https://doi.org/10.1002/advs.201400020

[S9] T. Ramireddy, T. Xing, M. M. Rahman, Y. Chen, Q. Dutercq, D. Gunzelmann, A. M. Glushenkov. Phosphorus–carbon nanocomposite anodes for lithium-ion and sodium-ion batteries. J. Mater. Chem. A **3**(10), 5572-5584 (2015). https://doi.org/10.1039/C4TA06186A

[S10] Y. Zhang, L. Wang, H. Xu, J. Cao, D. Chen, W. Han. 3D chemical cross-linking structure of black phosphorus@CNTs hybrid as a promising anode material for lithium ion batteries. Adv. Funct. Mater. **30**(12), 1909372 (2020). https://doi.org/10.1002/adfm.201909372
